# Sound-based assembly of a microcapillary network in a saturn-like tumor model for drug testing

**DOI:** 10.1016/j.mtbio.2022.100357

**Published:** 2022-07-12

**Authors:** Nicola Di Marzio, Preeta Ananthanarayanan, Anne Géraldine Guex, Mauro Alini, Chiara Riganti, Tiziano Serra

**Affiliations:** a AO Research Institute Davos, 7270 Davos, Switzerland; b Department of Health Sciences, Università Del Piemonte Orientale (UPO), Novara, Italy; c Department of Oncology, University of Torino, 10126, Torino, Italy; d Inter-departmental Centre “G. Scansetti” for Studies on Asbestos and Other Toxic Particulates, University of Torino, 10126, Torino, Italy

**Keywords:** Biofabrication, Acoustic, Microcapillary network, Tumor microenvironment, Malignant pleural mesothelioma, 3D models

## Abstract

The tumor microenvironment (TME), consisting of extracellular matrix, proteins, stromal cells, and a vascular system, is reported to have a key role in cancer progression and prognosis. Thereby, the interaction between the vascular network and tumor mass is an important feature of the TME since the anticancer agents which are delivered to the TME can trigger the vascular response and influence the therapeutic outcome of the treatment. To identify and develop new therapeutic strategies, 3D *in vitro* models that recapitulate the complexity of the TME are urgently needed. Among them, vascularized tumor models are a promising approach, allowing to target tumor angiogenesis and reduce tumor growth. By using sound patterning, cells can be condensed locally into highly reproducible patterns through the action of mild hydrodynamic forces. Here, we use a soundwave-driven cell assembly approach to create a ring-shaped microcapillary network in fibrin hydrogel. Then, we generate a 3D vascularized tumor model by combining a tumor heterotypic spheroid, consisting of fibroblasts and Malignant Pleural Mesothelioma (MPM) cells, with the surrounding vascular ring. Based on its shape, we name it Saturn-like vascularized Tumor Model (STM). The growth of the microcapillary network is monitored over time by fluorescence imaging. The area covered by the microcapillary network, and its continuous increase in presence of the heterotypic tumor spheroid was monitored. Interestingly, this effect is enhanced when treating the STM with the anticancer agent Cisplatin. Overall, we show the use of sound patterning as a fast and cell-friendly approach to spatially organize and condense cells, to generate a 3D *in vitro* platform from which simple readouts of drug tests can be extracted by image analysis, with the potential to provide a model system for tailored tumor therapy.

## Highlights

• Reproducible ring-shaped microcapillary networks were created by sound assembly.
• Ring microcapillary network and tumor spheroid formed the Saturn-like tumor model.
• ‘Radial profile’ analysis was used to monitor the ring microcapillary networks.
• Growth of the microcapillaries was modulated by tumor spheroid and anticancer drug.
• Anticancer drug upregulated pro-angiogenic related genes in the tumor spheroid.

## Graphical abstract

**Figure:**
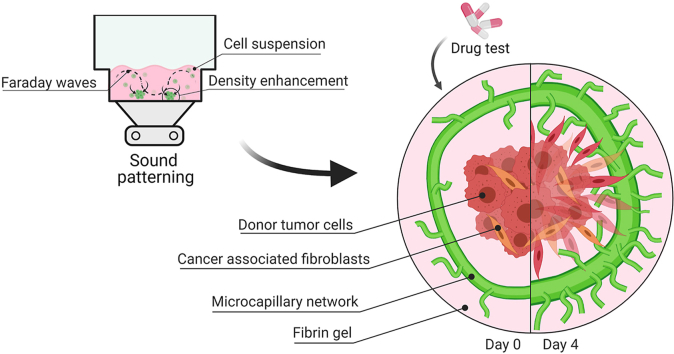

## 1 Introduction

With a growing aging population, the prevalence of cancer increases, and more advanced and personalized 3D *in vitro* models are required to improve the screening process [1,2]. Among the different cancer, Malignant Pleural Mesothelioma (MPM) is a highly aggressive tumor, caused by exposure to asbestos, with a slow progression and delayed diagnosis. Since most MPM are diagnosed at a late-stage, systemic therapy, based on cisplatin and pemetrexed derivatives, is the first choice. However, the rate of chemotherapy success is low, leading to a median survival of less than 12 months [[3], [4], [5], [6]]. One of the main reasons of the chemotherapy inefficacy is the poor drug delivery within the tumor bulk [7], dictated by the complex and heterogenous tumor microenvironment (TME). MPM TME consists of the extracellular matrix (ECM) and a variety of stromal cells, such as cancer-associated fibroblasts (CAFs), endothelial cells, pericytes, and immune cells, which systematically promote tumor progression [8]. The formation of the TME is mediated by MPM cells which secrete various components like collagen type IV, laminin and fibronectin that facilitate cell-cell communication and chemotaxis [8]. In addition, MPM bulk is often hypoxic: this condition increases the resistance to chemotherapy, by activating the Hypoxia-Induced Factors (HIFs), which promotes cell survival, migration, epithelial-mesenchymal transition, and drug efflux from MPM cells [[9], [10], [11]]. Vascular endothelial growth factor (VEGF), a well-known target of HIF [8,12,13], promotes the formation of a vascular network which supports tumor growth and metastatic progression [11,14]. Hence, there is a great need to develop *in vitro* platforms which effectively mimic components of the MPM TME to study the multi-factorial processes that may impair the efficacy of anticancer therapies and to test new drugs or combinations overcoming this limitation [11].

Several direct and indirect co-culture models using Transwell filters have been adopted to study the crosstalk between cancer cells and endothelial cells [[15], [16], [17]], whereas different approaches have been proposed to transition from simple 2D co-cultures towards more complex 3D models. Although state of the art microfluidic systems have shown good perfusion of randomly formed microcapillary networks [18,19] and availability for high throughput platforms [[20], [21], [22], [23], [24], [25], [26]], emerging biofabrication technologies have gained increasing popularity thanks to their ability to control the spatial organization of the TME components [2]. Other cell assembly strategies make use of magnetically or acoustically driven approaches to locally assemble biological components [1,27]. Among them, sound-induced morphogenesis (SIM) exploits low-frequency waves to generate hydrodynamic drag forces which locally assemble cells or cell aggregates within fluids [28]. Upon application of acoustic vibrations at low-frequency (<200 ​Hz), Faraday waves are generated at the liquid-air interface of a macroscale container and cells or microparticles are locally positioned beneath nodal positions of the standing wave [29], generating precise patterns in a simple, fast, and contactless way. SIM cell patterning is a cell-friendly and cell-sparing process which results in tightly packed cells in 3D. It enhances cell-cell contacts, cross-talk and anisotropic organization which has been shown to promote myotube formation [30], angiogenesis [31], neural differentiation [32], or cardiomyocyte differentiation [33].

**Fig. 1:**
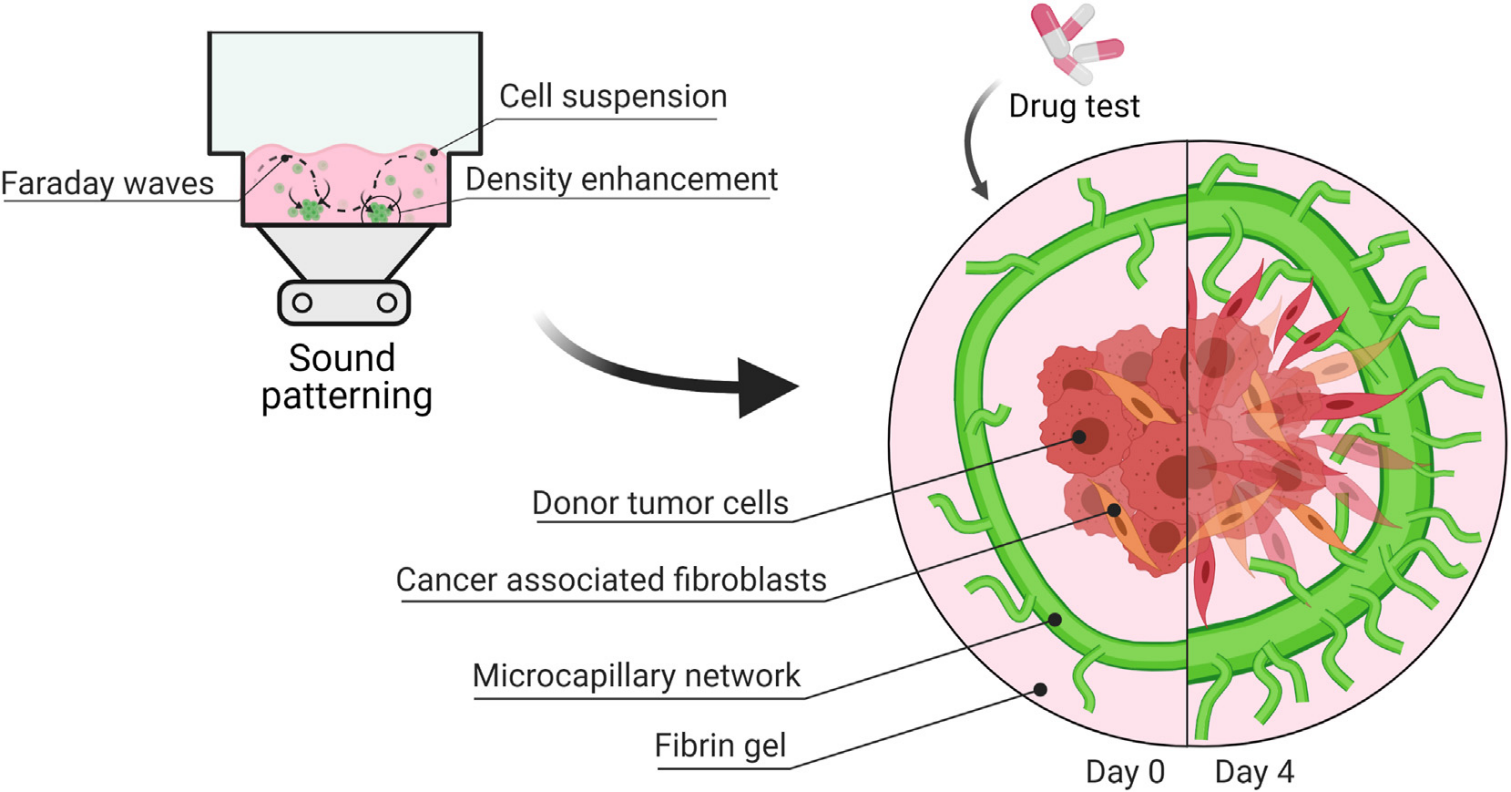
The tumor microenvironment is simulated in the Saturn-like 3D tumor model (STM). Firstly, (A) Sound patterning is used for shaping reproducible rings of GFP-HUVEC and human pericytes in fibrin within a commercially available multi-well plate. The cells self-assemble in a microcapillary network while retaining the ring architecture. Secondly, (B) a tumor heterotypic spheroid is embedded in fibrin gel and added onto the microcapillary network bed. The basal bed recapitulates the hierarchically organized microcapillary network stimulated by an establishing tumor in its proximity. (C) The model's microvascular morphology can be monitored, and the readouts could serve in drug testing applications to predict patient tailored treatments.

With increasing complexity of *in vitro* systems, the design of the models should facilitate simple and fast methods to analyze the different tissue components. The ability to individually observe and access the different tissue components remains a challenge. Here, using SIM, we developed an *in vitro* tumor model consisting of a microcapillary ring with a superimposed tumor spheroid in fibrin. Due to its structural appearance, the model is referred to as the Saturn-like Tumor Model (STM) (Fig. 1). This STM recapitulates important aspects of the TME and provides a versatile platform to evaluate new treatment strategies. Specifically, the symmetric, ring-shaped microcapillary enabled simple and quantitative image analysis to quantify the development over time in presence or absence of a tumor spheroid. In the course of validating this model, we assessed the response to anticancer treatment, composed of a first-line chemotherapeutic agent, Cisplatin, alone or in combination with a recently approved phase III anti-angiogenic drug, the *anti*-VEGF Bevacizumab.

Based on established image analysis, the microcapillary network in the TME model can be observed over time, providing non-destructive quantitative information on changes in response to the presence of the MPM tumor spheroid or anti-cancer treatments. Additional RT-PCR analysis on angiogenesis-related genes revealed the upregulation of several proangiogenic genes in the MPM cells upon chemotherapeutic treatment. We are thereby providing a new platform that will significantly contribute to a better understanding of tumor – vascular interactions and allow to evaluate new therapeutic approaches that target the TME.

## 2 Materials and methods

All chemicals were purchased from Sigma-Aldrich, Switzerland, and used as received without any further purification unless stated differently. Cell culture flasks and plastic ware were purchased from Techno Plastic Products AG (TPP, Switzerland). Cell culture media and supplements related to cell culture were from Gibco, distributed via ThermoFisher Scientific, Switzerland.

### 2.1 Fibrin gel preparation

Fibrinogen stock solutions (64% protein, 90% clottable fraction) were prepared at concentrations of 15 ​mg⋅mL^−1^ in phosphate buffered saline (PBS) and incubated for 1 ​h at 37 ​°C. The solutions were sterile-filtered using 0.2 ​μm pore filters, aliquoted, and stored at −20 ​°C. A 100 IU·mL^−1^ thrombin stock solution was prepared by reconstituting lyophilized thrombin from human plasma in a solution of 1.1% (w/v) NaCl and 2 ​mM CaCl_2_ and stored at −20 ​°C. To create the fibrin gel, working solutions of fibrinogen (5 ​mg⋅mL^−1^ in 1.1% (w/v) NaCl) and thrombin (0.5 IU⋅mL^−1^ in appropriate medium) were mixed in equal volumes on ice to obtain a final concentration of 2.5 ​mg⋅mL^−1^ fibrinogen and 0.25 IU⋅mL^−1^ thrombin. To assess the response to treatments, the respective drugs were added in a 2x concentration to the thrombin-medium solution and subsequently mixed 1:1 (v/v) with the fibrinogen solution to reach the final treating concentration (see section 2.6 for more details).

### 2.2 Cell culture

Green Fluorescent Protein-expressing Human Umbilical Vein Endothelial Cells (GFP-HUVEC, cAP-0001GFP) were purchased from Angio-Proteomie, USA, and expanded in Complete Endothelial Cell Growth Medium (EGM-2, Lonza Group AP, Switzerland) in pre-coated culture flasks (Quick Coating Solution, Angio-Proteomie, USA). Human pericytes from placenta (hPC-PL, PromoCell GmbH, Germany) were expanded in complete Pericyte Growth Medium 2 (PromoCell GmbH, Germany). Human normal lung fibroblasts (MRC-5, ATCC® CCL-171™) were cultured in Minimum Essential Medium, alpha modification (α-MEM, Gibco) with 2 ​mM l-Glutamine, supplemented with 10% (v/v) fetal bovine serum (FBS, Gibco) and 1% (v/v) penicillin/streptomycin (P/S). The primary human epithelioid Malignant Pleural Mesothelioma (MPM) cells, obtained after diagnostic thoracoscopies, were kindly provided by the Biologic Bank of Malignant Mesothelioma, S. Antonio e Biagio Hospital (Alessandria, Italy; approval by the local ethical committee: #9/2011) [34]. MPM cells were expanded in Ham's F-12 ​cell growth medium supplemented with 10% (v/v) FBS and 1% (v/v) P/S. For all cell types, cell culture media were refreshed every 48 ​h, cells were passaged by trypsinization at 90% confluency. Cell culture was maintained at 37 ​°C in a humidified incubator at 5% CO_2_.

### 2.3 Tumor spheroid formation

**Fig. 2:**
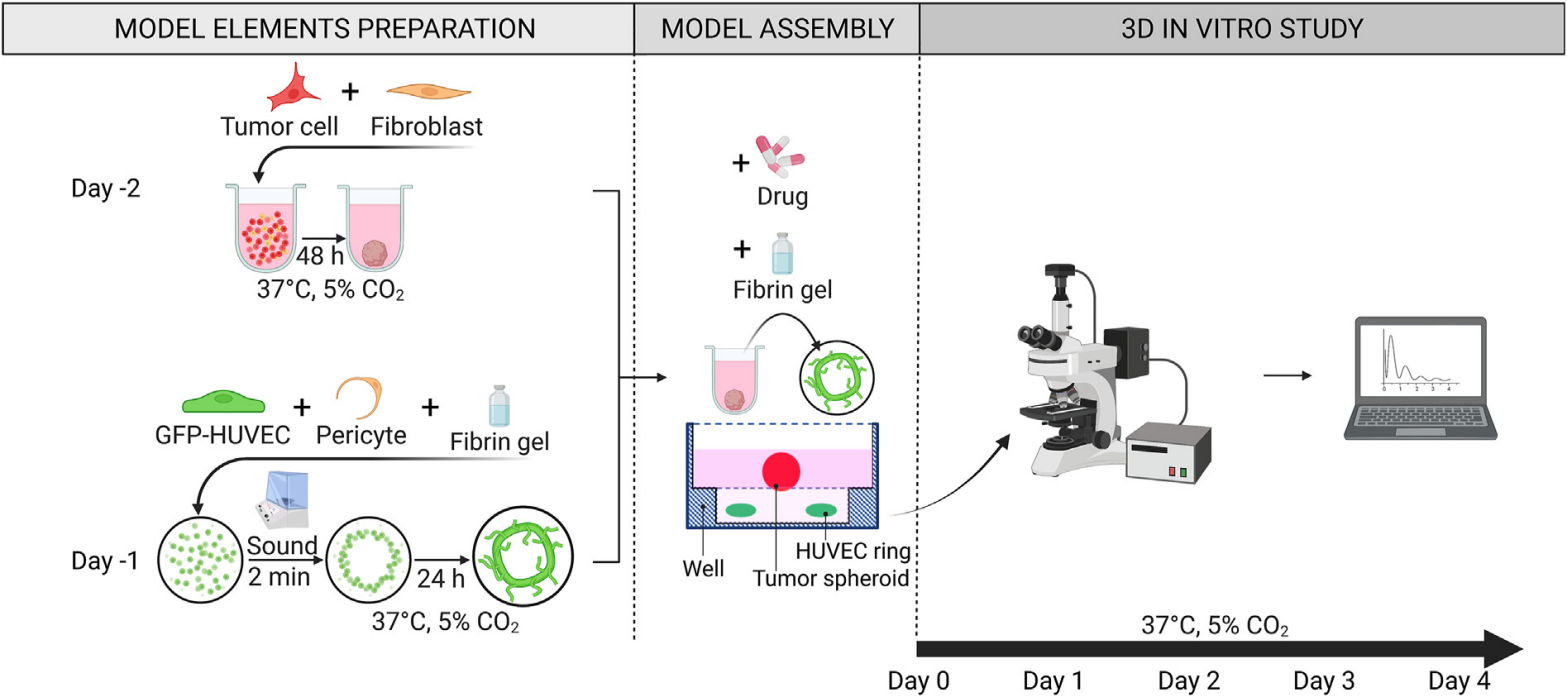
Saturn-like 3D Tumor Model fabrication and experimental workflow. At day −2 the Malignant Pleural Mesothelioma (MPM) and human fibroblast (MRC-5) cells are collected and mixed at a ratio of 10:1 to prepare a heterotypic spheroid in a 96-well ultra-low adhesive plate. At day −1, green fluorescent expressing human umbilical vein endothelial cells (GFP-HUVEC) and human pericytes from placenta (hPC-PL) are collected and mixed at a ratio of 20:1 in a single cell suspension made in fibrin and dispensed into the inner compartment of the well. Before gelation, Faraday waves are generated at the liquid-air interface and sound-induced hydrodynamic forces condense the cells in a ring pattern in less than 2 ​min. At day 0, the STM is assembled by adding the heterotypic spheroid to the microcapillary ring and different drugs are added. The assembled STM is kept in culture under treatment for 4 days and readouts are acquired daily from fluorescence microscopy images.

At day −2 (Fig. 2), heterotypic tumor spheroids were formed by spontaneous assembly of MPM cells and MRC-5 in low adhesion well plates. The MPM and MRC-5 were seeded in a Nunclon™ Sphera™ 96-well U-bottom ultra-low attachment microplate (ULA Nunclon Sphera, ThermoFisher Scientific) in a ratio of 10:1 (MPM:MRC-5; 40.000:4.000 ​cells per well) and cultured in respective mixed media (1:1, (v/v)). The plate was incubated at 37 ​°C in a 5% CO_2_ humidified atmosphere for 48 ​h to facilitate self-assembly into the heterotypic tumor spheroids. Spheroid diameters were measured based on bright field microscopy images for a total of n ​= ​30 spheroids.

### 2.4 Sound patterning of microcapillary rings in a multi-well plate

The sound patterning setup and methodology was previously established in our group [28]. Briefly, a sound patterning system also known as SIM device (CymatiX, mimiX Biotherapeutics, CH) was used to generate the 3D microcapillary ring. The SIM device consists of a sound-vibration plate, sample clamping system and control system for setting frequency and amplitude of the sound vibration. After loading of the patterning chamber with the desired cell-laden hydrogel precursor, the sample is placed onto the sound-vibration plate, fixed with the clamping system and the vibration is applied for the desired time.

At day −1 (Fig. 2), HUVEC and hPC-PL were trypsinized and mixed in a cell suspension at a ratio of 20:1 HUVEC:PC-PL. The mixed cell suspension was used to prepare a thrombin-EGM-2-cells suspension (0.6, 1, or 1.5⋅10^6^ ​cells⋅mL^−1^) and kept on ice until use. Fibrinogen (5 ​mg⋅mL^−1^) and thrombin-EGM-2-cells suspension were mixed in equal volumes and 7.5 ​μL of it was loaded on ice into the inner well-compartment of the IBIDI μ-Slide Angiogenic plate (IBIDI, Germany). In a room with a temperature-control set at 16 ​°C, the μ-Slide was then transferred onto the sound-vibration plate of the SIM. By applying a sound vibration with characteristic frequency of 54 ​Hz and 0.2 ​g vertical acceleration, the ring pattern was created within 2 ​min. After fabrication, the μ-Slide was left on the machine for 1 more minute to permit fibrin gelation and pattern stabilization. The μ-Slide was then incubated at 37 ​°C for 2 ​min to further progress gelation of the fibrin gel. The procedure was repeated for the remaining wells of the μ-Slide. The upper well was then filled with 50 ​μL EGM-2 growth medium and maintained at 37 ​°C in a 5% CO_2_ humidified atmosphere. The same cell suspension of HUVEC:PC-PL in fibrin was also seeded into the multi-well labware without the application of sound for a randomly organized capillary network formation.

### 2.5 Saturn-like 3D tumor model (STM) assembly

At day 0 (Fig. 2), single tumor spheroids were gently collected from the ULA 96-well plate and added into each well of the μ-Slide on top of the previously assembled μ-capillary ring (named as P ​+ ​S condition) or onto the randomly assembled μ-capillary network (named as R ​+ ​S condition). The growth medium of the cancer spheroid was first discarded from its well, the spheroid was embedded in 20 ​μL of fibrin (w/or w/o drug), removed from the well and placed on top of the μ-capillary network. The μ-Slide with the final assembled model was then incubated at 37 ​°C to complete fibrin gelation. 30 ​μL of EGM-2 growth medium (w/or w/o drug) was added to each well and the μ-Slide was maintained at 37 ​°C in a 5% CO_2_ humidified atmosphere for 4 days with daily medium change (w/or w/o drugs).

### 2.6 Chemotherapeutics treatment preparation and administration to the model

Cisplatin (cis-Diamineplatinum (II) dichloride) (Sigma-Aldrich, Italy), solubilized at 50 ​mM solution in Dimethylsulfoxide (DMSO), was administrated at a concentration of 50 ​μM; Bevacizumab (Selleckchem, Houston, TX), purchased in solution at 5 ​mg⋅mL^−1^, was administrated at a concentration 10 ​μg⋅mL^−1^, with daily renewal. From day 0 onwards, 30 ​μL drug-EGM-2 solutions were dispensed on top of the fully built and jellified construct. In addition to the drug-treated culture medium, drug-infused fibrin gel was used for embedding the cancer spheroid and avoid diffusion gradients.

### 2.7 Imaging of the STM over time

The microcapillary layer of the STM was monitored from day −1 (ring pattern formation) until day 4 (end point) and characterized based on image analysis (Fig. 2). Bright field images of the model and fluorescent images of the GFP-expressing HUVEC were acquired using a Zeiss LSM800 confocal microscope equipped with a CCD camera (Axiocam 506 color). Daily, the 3D μ-capillary network volume was imaged with a 5x objective. A scanning field of 3 ​× ​3 tiles was used, and 225 ​μm thick Z-stacks were acquired for each tile with a slice thickness of 17.3 ​μm. The corresponding imaged area and volume in the well was about 13.8 ​mm^2^ (3.58 ​mm ​× ​3.58 ​mm) and 3.1 ​mm^3^. From the Z-stacks, the Z-projections of the tiled images were generated and used for post processing analysis. Z-projection and image analysis of the fluorescent images were performed using ImageJ software (NIH, Bethesda, MD, USA).

### 2.8 μ-capillary ring pattern readouts from the ‘radial profile’ analysis

The geometry and reproducibility of the HUVEC-hPC-PL patterns were measured from the images acquired right after sound patterning. An ImageJ macro was created to extract the morphological characteristics of the pattern from the Z-projected images of the capillary network. The ImageJ's plugin “Radial profile” was used to measure diameter and thickness of the microcapillary ring. Briefly, the ImageJ's plugin measured the intensity values along the radius of a region of interest (ROI) placed concentrically with the pattern, the radius was spun over a 360°-degree and the intensity values were integrated and plotted against the radial distance from the center of the ROI. The coordinate of the signal's peak and its full width at half maximum were calculated with a MATLAB script and were identified as the ring's radius and thickness. The radial profile analysis was also used to quantify the drug response. The area under the curve (AUC) of the signals and the peak intensity level were extracted from the signals at the end of the study. A total of 3 images per experimental group for 3 individual experimental repeats were analyzed and the results plotted in the corresponding graph.

### 2.9 Tracking of μ-capillary network evolution

Cellular assembly and growth of the μ-capillary network was evaluated by measuring the total area of the GFP-HUVEC network daily throughout the culture period (day −1 to day 4). An ImageJ macro was created to obtain the Z-projection of the Z-stack acquired from the GFP-channel, a binary mask of the network was generated, and the ImageJ's function “Analyze particles” was used to quantify the total area. Data were plotted as relative increase to the GFP-area measured on day −1. A total of 3 images per experimental group for 3 individual experimental repeats were analyzed and the results plotted in the corresponding graph.

### 2.10 RNA extraction and PCR-array for the transcriptomic profile

In a 96-well plate the 3D model system was recapitulated as 3D multicellular culture in order to have access to sufficient biological material to use in the downstream analysis. The microcapillary component was randomly seeded in each well as 3D HUVEC ​+ ​Pericytes culture (20:1 HUVEC:Pericyte) in fibrin (fibrinogen 2.5 ​mg⋅mL^−1^, Thrombin 0.25 U⋅mL^−1^). After 24 ​h, the tumor spheroid (MPM317:MRC-5 10:1, 44000/spheroid in culture for 48 ​h in ULA 96-well plate) was embedded in fibrin gel and added into each well. At this stage (day 0) the drug treatments were administrated and refreshed daily until day 4. Drugs solutions were prepared in fresh EGM-2 medium and Cisplatin 50 ​μM, Bevacizumab 10 ​μg⋅mL^−1^, Cisplatin 50 ​μM ​+ ​Bevacizumab at 10 ​μg⋅mL^−1^ or EGM-2 alone were used for the treatments of the 3D constructs. In addition to the tumor-microcapillary coculture, tumor spheroid and microcapillary alone were cultured in fibrin and separately treaded with the same drug formulations. At day 4, 150 ​μL of TRIzol™ Reagent (ThermoFisher Scientific, Switzerland) were added into each well and incubated for 10 ​min, the biological material of 3 wells from each condition was pooled together for the RNA extraction. RNA was extracted with RNeasy Mini Spin Columns (Qiagen, Hilden, Germany). 100 ​ng RNA were retro-transcribed with the iScript™ cDNA Synthesis Kit (Bio-Rad Laboratories). The PCR arrays were performed on 0.1 ​μg cDNA, using the PrimePCR PCR Array (Bio-Rad Laboratories), as per manufacturer's instructions. The Gene Expression Quantification software (Bio-Rad Laboratories) was used to assess relative gene expression levels, normalizing the expression of each gene of interest on the mean expression of a pool of housekeeping genes (*B2M*, *GAPDH*, *HPRT1*, *TBP*, *RLP0*, *GUSB*) present in the array.

### 2.11 Statistical analysis

In all experiments, analyses for statistically significant differences between multiple conditions were carried out using one-way ANOVA test, the homoscedasticity was assumed due to the hypothesis of equal or similar network status at the beginning of the treatment among the different groups. The treatment groups were compared with the control group (EGM-2) at each time point or specifically indicated and Dunnett's test was applied. All data in the graphs are reported as mean value of three experiments independently reproduced, with standard deviation as error bars or dotted line. Statistically significant differences were considered with *P* ​< ​0.05 (∗*P* ​< ​0.0332, ∗∗*P* ​< ​0.0021, ∗∗∗*P* ​< ​0.0002, ∗∗∗∗*P* ​< ​0.0001). Number of samples are indicated in the figure legends with N ​= ​number of individual experiments and n ​= ​number of technical repeats.

## 3 Results

### 3.1 Sound patterning generates reproducible ring-shaped microcapillary networks

**Fig. 3:**
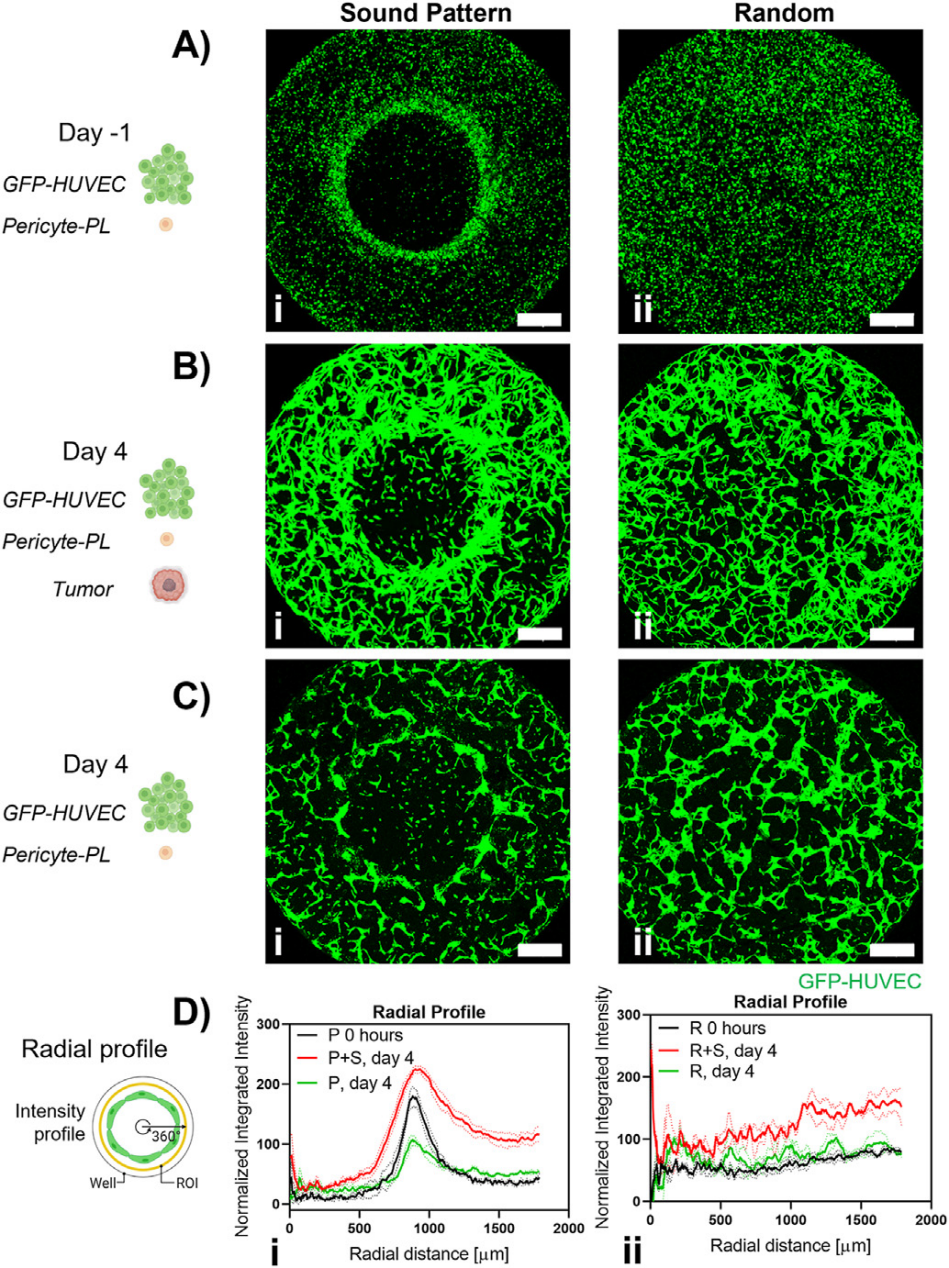
The sound patterned microcapillary ring can be monitored with ‘Radial profile’ analysis in contrast to a randomly generated network. (A,i) The diameter of the ring-shaped cell condensate after sound patterning is 1781 ​± ​142 ​μm (mean ​± ​SD, N ​= ​4 and n ​= ​25). In contrast, (A,ii) the same cell suspension is statically seeded in fibrin without applying the sound waves and showed homogeneous cell distribution within the well. After 4 days in coculture with the tumor spheroid, (B,i) the microcapillary ring has presented higher ring continuity and increased ring thickness compared to (C,i) the microcapillary ring network without tumor spheroid. (B,ii) Although a macroscopic network organization is not observed in the randomly formed microcapillary network, it has developed more in presence of the tumor spheroid compared to (C,ii) the network development in absence of it. (D,i) The ‘Radial profile’ signal obtained shows a peak which is associated with the presence of the ring pattern; the ‘Radial profile’ was used to monitor the microcapillary ring evolution over time in presence (P ​+ ​S) or absence (P) of the tumor spheroid. The peak becomes wider and higher in presence of the tumor spheroid and thinner and smaller in absence of the cancer mass. Continuous lines indicate mean values, dotted lines indicate SD. (D,ii) No characteristic peak appears in the ‘Radial profile’ signal acquired from a randomly formed microcapillary network w/ (R ​+ ​S) or w/o (R) tumor spheroid. Scale bars 500 ​μm.

The HUVEC-Pericytes (hPC-PL) cell suspension in fibrin was patterned in a ring shape in each well of the IBIDI μ-Slide Angiogenic plate. The obtained ring made up of condensed cells (Fig. 3, Ai; Fig. S1, A) had a diameter of 1781 ​± ​142 ​μm and a thickness of 416 ​± ​124 ​μm (mean ​± ​SD, 4 independent experimental repeats with minimum n ​= ​25 patterns per experiment), while in absence of sound vibration the cells were distributed homogeneously (Fig. 3, Aii). HUVEC cells self-assembled into a microcapillary network while retaining the ring-pattern organization throughout the 5 days of culture (Fig. 3, Bi, Ci). The cell concentration in the fibrin was optimized before creating the patterns. Fibrin cell concentrations of 0.5, 1 or 1.5⋅10^6^ ​cells⋅mL^−1^ were tested and the condensation of cells in a ring pattern was achieved with all cell concentrations (Fig. S2). 1.5⋅10^6^ ​cells⋅mL^−1^ resulted in the most pronounced hierarchically organized microcapillary network (continuous thicker ring microcapillary connected to a highly branched external microcapillary network) and was therefore chosen for this study. The fibrin around the microcapillaries was colonized by the pericytes and phase contrast images also showed the alignment of pericytes along the contour of the GFP-HUVEC capillaries (Fig. S1, B).

### 3.2 Tumor spheroids are combined with the sound patterned microcapillary network to create the STM

Single multicellular tumor spheroids with a diameter of 926 ​± ​30 ​μm (mean ​± ​SD, n ​= ​30) were generated after 48 ​h of culture in the ULA 96 well plate. The seeding ratio and cell number per spheroid was optimized to match a spheroid size roughly comparable to the inner radius of the microcapillary ring. At day 0, the multicellular tumor spheroid was placed on top of the HUVEC ring pattern and the STM model was finally assembled. The schematic representation of the 3D structure of the model is reported in Fig. 2. The final construct resulted in 2 layers, one containing the microcapillary ring and one the tumor spheroid; the distance between the spheroid's plane and the microcapillary pattern plane was 76 ​± ​36 ​μm (n ​= ​6) and the overall model's thickness was 420 ​± ​18 ​μm (n ​= ​6).

### 3.3 The changes in morphology of sound patterned microcapillary network can be monitored with ‘radial profile’ analysis in contrast to a randomly generated network

Due to the radial symmetry of the ring geometry, the ‘Radial profile’ image analysis technique could detect the presence of the HUVEC pattern with a clear peak in its output signal. The pattern of cells in the images could be detected at the patterning day (day −1) and was still detectable at the end of the study (day 4) (Fig. 3, Di). The result of ‘Radial profile’ analysis was also susceptible to the presence or absence of the tumor spheroid. In fact, when the detected peaks were compared (Fig. 3, Di), it was observed that the peak in the signal was increased twofold and wider when the microcapillary ring was cultured in presence of the tumor spheroid (Fig. 3, Bi) than the microcapillary network alone (Fig. 3, Ci). The ‘Radial profile’ analysis on the randomly organized microcapillary network did not show a characteristic peak (Fig. 3, Dii) that would indicate the presence of any anisotropy or geometry. The presence of the cancer spheroid was characterized by an overall increase in signal intensity (Fig. 3, Bii), compared to randomly organized network cultured alone for the same period of time (Fig. 3, Cii).

### 3.4 The AUC of the ‘radial profile’ signal correlates with the response of the STM to drug treatments

**Fig. 4:**
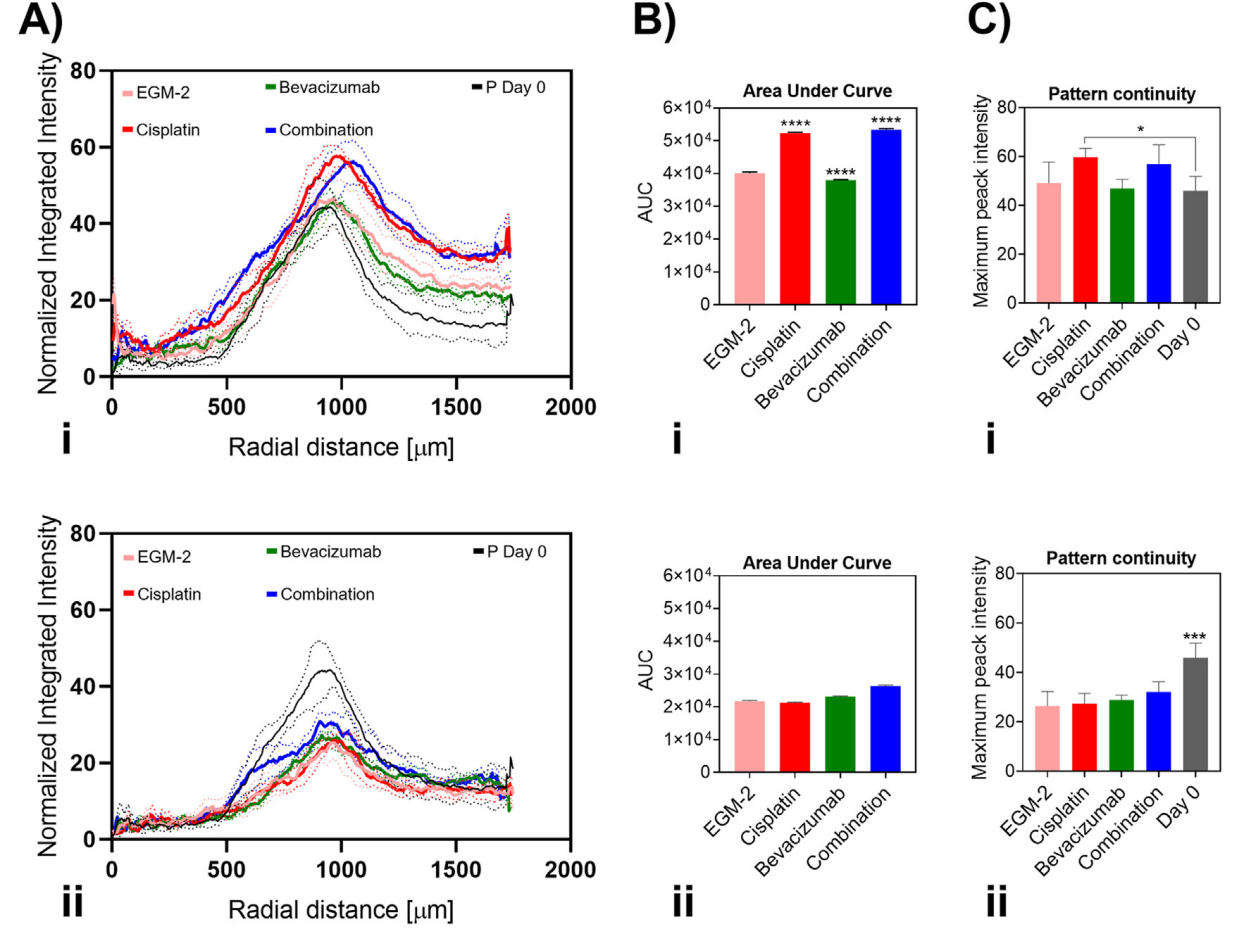
The ‘Radial profile’ readouts correlate with the response of the STM to drug treatments. The STM model's response to drug is tested with anticancer (Cisplatin 50 ​μM), antiangiogenic (Bevacizumab 10 ​μg⋅mL^−1^) drugs and their combination (at similar concentrations). (A) The ‘Radial profile’ analysis measures different ring microcapillary morphologies when the model is treated with different drugs. (A,i) Significant differences in the signal morphology is observed in presence of the tumor spheroid, whereas (A,ii) no detectable difference is observed without presence of the tumor spheroid. Continuous lines indicate mean values, dotted lines indicate SD. (B,i) Quantifying the area under the curve (AUC) of the radial profile signal reveals 30% more AUC in the groups treated with Cisplatin compared to control (EGM-2 treated group) and (B,ii) no detectable difference is measured in absence of tumor spheroid in the system. The maximum peak intensity value is used as measure of the ring microcapillary network continuity, it is observed that in presence of tumor spheroid (C,i) the ring pattern continuity is also preserved (intensity peak higher than day 0). In contrast, (C,ii) the ring pattern in absence of tumor spheroid has lost its continuity and became a disaggregated network (intensity peak lower than day 0). N ​= ​3 and n ​= ​3.

The response of the microcapillary ring within the STM to different drug treatments was detectable by the ‘Radial profile’ analysis (Fig. 4, Ai). Significant differences were measured only when the microcapillary ring was cocultured with the tumor spheroid. In particular, when the STM was treated with Cisplatin or with Cisplatin ​+ ​Bevacizumab the AUC calculated at day 4 increased by 30% and 33%, respectively, compared to EGM-2 condition (P ​< ​0.05). Whereas, after the Bevacizumab treatment the AUC decreased by 5% compared to EGM-2 (P ​< ​0.05) (Fig. 4, Bi). In absence of the tumor spheroid the AUC did not highlight any significant differences among the treated groups (Fig. 4, Bii). Additionally, the continuity of the ring pattern in response to different culture conditions was extracted from the ‘Radial profile’ analysis. The ‘Radial profile’ peak maximum intensity value was found to be sensitive to the discontinuity presented in a ring shape and demonstrated to be proportional to the continuity of the ring shape itself (Fig. S3). The continuity of the ring-shaped microcapillary network was measured at the end of the study (day 4) and compared to the continuity of the ring on day 0. In presence of the tumor spheroid, there was no statistical difference detected in the microcapillary ring continuity (Fig. 4, Ci), whereas in absence of the tumor spheroid, the continuity of the ring microcapillary network was impacted and all the treatment conditions presented statistically lower pattern continuity compared to their status at day 0 (Fig. 4, Cii).

### 3.5 The presence of the spheroid and anticancer treatment promote microcapillary network growth over time

**Fig. 5:**
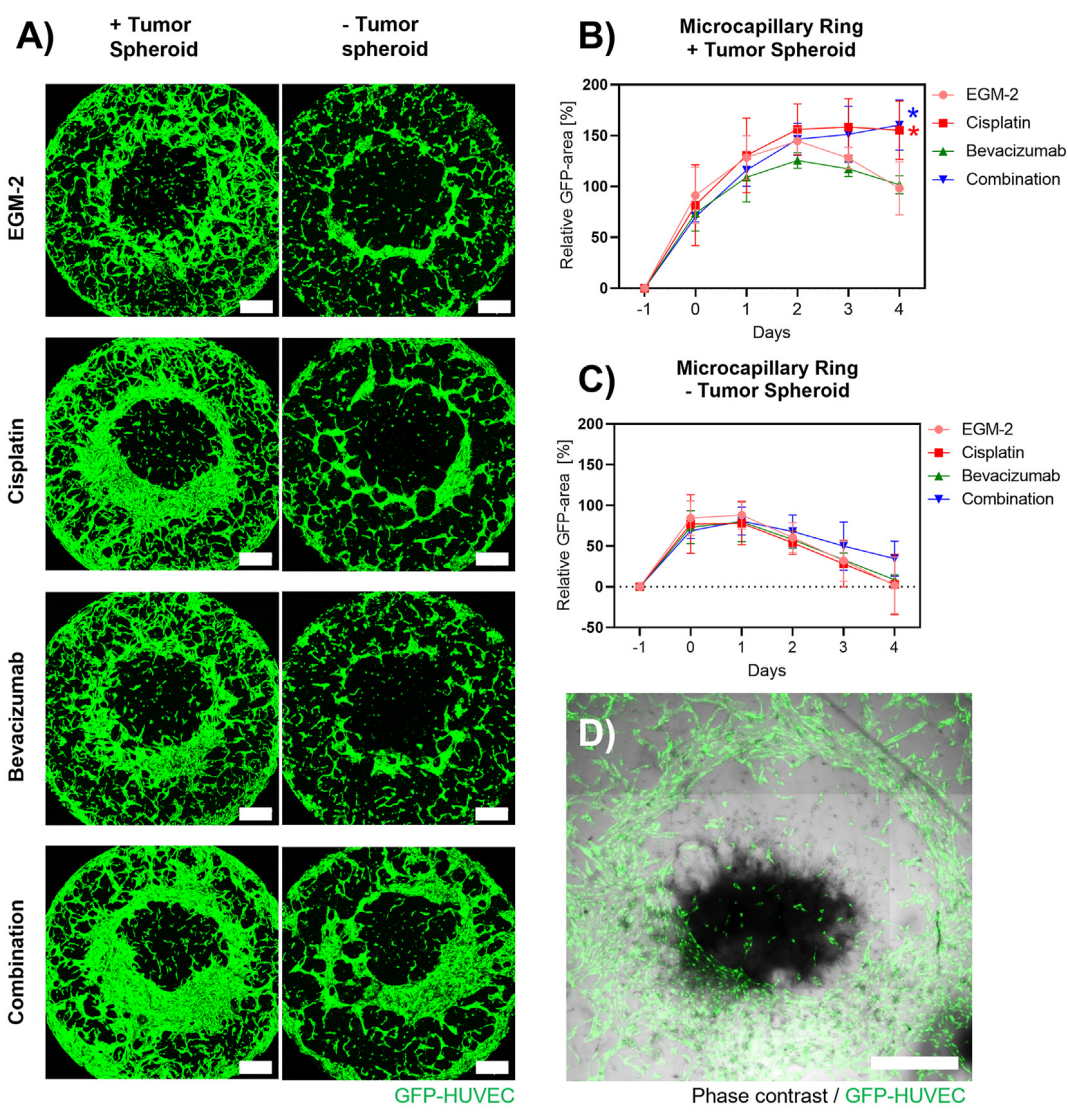
Changes in the microcapillary network growth over time. (A) At the study end point day 4, the difference in the ring microcapillary maturation with/without the spheroid in presence of different treatments is clearly visible. (B) The growth trend of GFP-HUVEC network's area over time indicates that the presence of the tumor spheroid is able to stimulate the microcapillary network growth. Additionally, when the STM is treated with the anticancer drug, Cispatin (50 ​μM) and Bevacizumab (10 ​μg⋅mL^−1^), alone or in combination, it induces the ring microcapillary network to cover 50% more area compared to the EGM-2 treated condition. While (C), the growth trend of the GFP-HUVEC network's area over time is inverted if the tumor spheroid is not added onto the microcapillary network and the drug treatments do not reveal a macroscopic change. (D) At day 4 in culture the established microcapillary network coexists with the tumor spheroid which is sprouting and invading fibrin (composite of GFP- and phase contrast-channel). N ​= ​3 and n ​= ​3. Scale bars 500 ​μm.

The microcapillary network developed differently in presence or absence of the tumor spheroid. Fig. 5(A) shows the Z-projected images of the GFP-channel Z-stack of the microcapillary layer of the model acquired at day 4 with or without the tumor spheroid for the different drug treatments. In presence of the tumor spheroid (left column) a pronounced microcapillary network can be observed, which is reduced in absence of it (right column). The GFP-positive area covered by the network in combination with the tumor spheroid had a daily growth until day 2 for all treatment groups (Fig. 5, B). The non-treated model (EGM-2) had the maximum area covered by the network at day 2, specifically demonstrating 145 ​± ​10% growth which decreased to 98 ​± ​21% at day 4. The Cisplatin-treated model had a similar growth up to day 2 with an increase of 156 ​± ​20% which, however, remained constant until day 4 (155 ​± ​23% significantly different compared to control, P ​< ​0.05). The Bevacizumab-treated model did not show significant differences in microcapillary growth compared to the non-treated model (125 ​± ​6% at day 2 and 102 ​± ​7% at day 4) and the combined treatments of Cisplatin ​+ ​Bevacizumab showed similar trends as the Cisplatin only -treated group (146 ​± ​13% at day 2 and 160 ​± ​20% at day 4, significantly different compared to control, P ​< ​0.05 ​at day 4) (Fig. 5, B). The network area had a growth rate with an opposite trend when the spheroid was not added to the system (Fig. 5, C). Indeed, in all the treatment conditions starting from day 1, a decrease in the GFP-positive area was observed and the network had continuous retraction until day 4. At day 4 the area of the network was comparable to the GFP-positive area measured at day −1, immediately after patterning and no significant difference was measured (Fig. 5, C).

### 3.6 PRC-array revealed overexpressed pro-angiogenic factors in response to cisplatin treatment

**Fig. 6:**
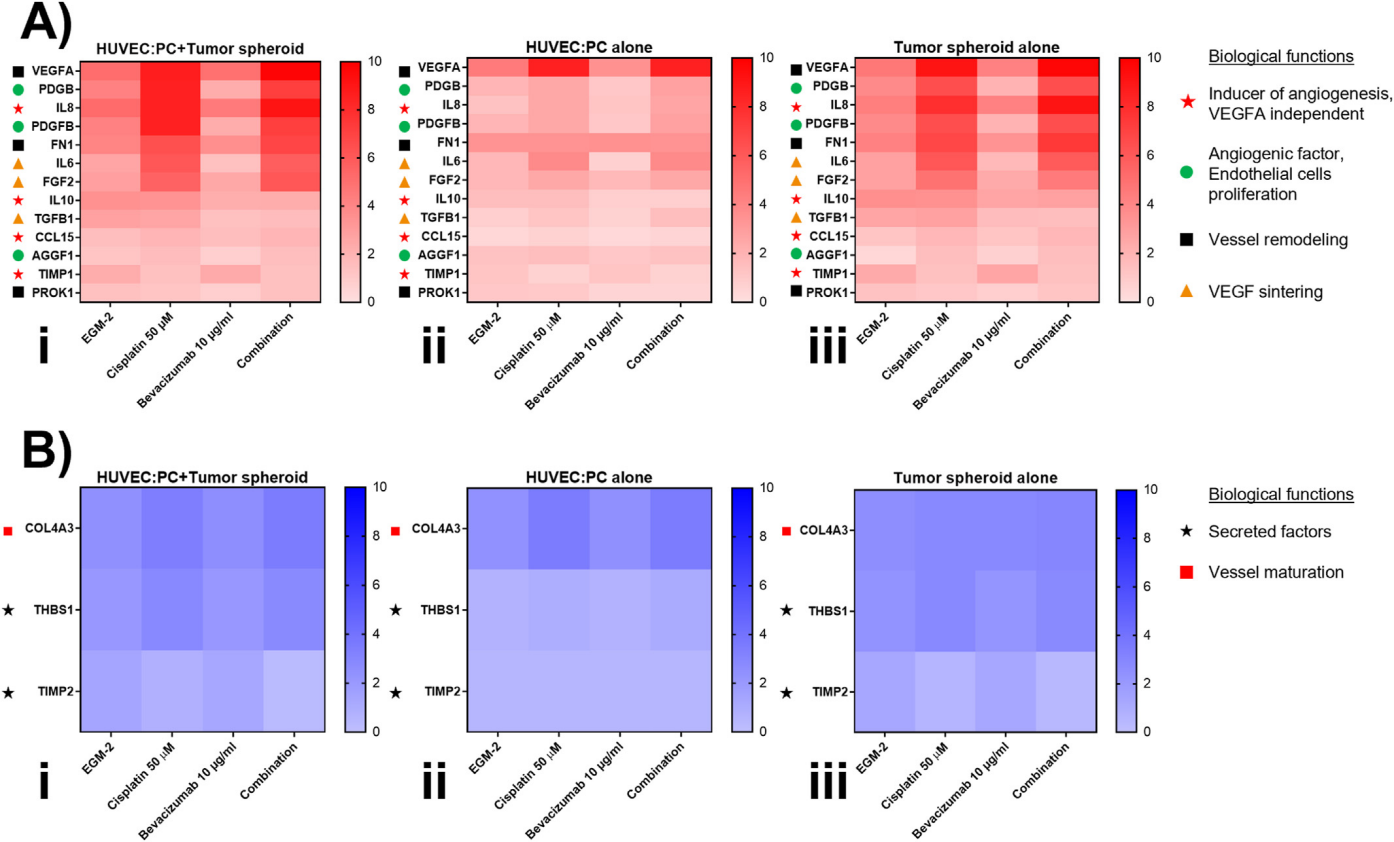
Summary of angiogenesis-related genes expressed in vascular bed, malignant pleural mesothelioma spheroid, and their coculture upon treatment. PCR-array results showed with heatmaps of (A) significantly variating pro-angiogenic and (B) anti-angiogenic genes from (Ai, Bi) HUVEC-pericytes and MPM tumor spheroid coculture, (Aii, Bii) HUVEC-pericytes alone, and (Aiii, Biii) MPM tumor spheroid alone. They were conditioned for 4 days with fresh medium (EGM-2), Cisplatin (50 ​μM), Bevacizumab (10 ​μg⋅mL^−1^) or their combination (Combination). The data were normalized to the mean expression of the housekeeping genes B2M, GAPDH, HPRT1, TBP, RLP0, GUSB (Gene Expression Quantification software, Bio-Rad Laboratories). The whole gene profile and statistical analysis are reported in the Supplementary Information Fig. S8 and Supplementary File 1. (N ​= ​3, n ​= ​2).

To better clarify the molecular circuitry underlying this phenomenon, we performed a targeted transcriptome profile of HUVEC/pericyte microcapillary network, MPM tumor spheroid and their coculture after treatment with Cisplatin, Bevacizumab or their combination (Fig. 6 & Fig. S8). Cisplatin alone significantly upregulates at transcriptional levels some proangiogenic factors involved in endothelial cells proliferation and sprouting, including VEGFA, VEGFA-synergizing factors (fibroblast growth factor 2, FGF2; interleukin 6, IL6) and VEGFA-independent soluble factors (platelet-derived growth factor β, PDGFB; chemokine (C–C motif) ligand 15, CCL15; tumor necrosis factor α, TNFα; interleukin 8, IL8; TIMP metallopeptidase inhibitor 1, TIMP1) in HUVEC/pericyte cells (Fig. 6, Aii). The same genes were upregulated in MPM spheroids, together with additional pro-angiogenic factors as angiogenic factor with G patch and FHA domains 1 (AGGF1) and fibronectin 1 (FN1) (Fig. 6, Aiii). Only two-antiangiogenic factors - collagen 4A3 (COL4A3) and thrombospondin 1 (THBS1) - were increased by Cisplatin, while the tumoral anti-angiogenic factor TIMP metallopeptidase inhibitor 2 (TIMP2) was down-regulated (Fig. 6, B). This transcriptomic signature, markedly pro-angiogenic, was reproduced in the coculture of the microcapillary with the MPM tumor spheroid, even to a higher extent, likely because genes of both endothelial and tumoral origin were up-regulated at the same time (Fig. 6, Ai,ii,iii). Compared to Cisplatin, Bevacizumab had few transcriptional effects. Interestingly, it significantly up-regulated IL6, synergizing with Cisplatin, while it down-regulated the pro-angiogenic factors PDGFB, interleukin 10 (IL10), prokineticin 1 (PROK1) and transforming growth factor β (TGFB). Moreover, Bevacizumab did not significantly modulate any anti-angiogenic factors (Fig. 6, B & Fig. S8). When used in combination, the effects of Cisplatin were predominant over Bevacizumab, because the transcriptome profile of endothelial cells, tumor cells and STM was superimposable to that of Cisplatin and markedly differed from that of Bevacizumab (Fig. 6 & Fig. S8). This experimental set suggests that the pro-angiogenic effects observed in the presence of Cisplatin, and not reversed by Bevacizumab, were due to the transcriptional activation of multiple pro-angiogenic genes, either VEGFA-dependent/synergizing or VEGFA-independent.

## 4 Discussion

The idea of the here presented work was to create a simple 3D *in vitro* model using sound patterning to create reproducible microcapillary networks which can be combined with a secondary biological element in a layer-by-layer approach. As a proof of concept, we generated a multicellular 3D model which mimics the microcapillary network of the MPM TME. Leveraging the mild hydrodynamic force of the sound waves, we managed to pattern a first layer of endothelial cells and pericytes, achieving highly reproducible spatial organization in less than 2 ​min within a commercially available multi-well plate (IBIDI μ-slide). In accordance with our previous findings [28], the pre-shaping of the fibrin-cell suspension with a single application of sound was sufficient to maintain the desired pattern of endothelial cells throughout the self-assembling process into a capillary network and during the culture days. The microcapillary ring network was created in fibrin which is a natural polymer frequently used for engineering microcapillaries as it is an optimal ECM-like matrix [35]. By operating the SIM device in a temperature-controlled environment, sound patterning was applied to the cell suspension before the fibrin was completely gelled. Moreover, sound patterning is a versatile process which could be smoothly translated to other ECM-like hydrogels as reported by Ren et al. [36] thereby expanding this process towards other applications in the field of tissue engineering. In addition to the spatial organization, sound patterning results in local cell condensation which has been shown to be relevant, in the vessel tissue engineering field, for the formation of a single tubular capillary rather than a branched capillary network [24]. Tuning the initial cell density and the stimulation time, sound patterning generated gradients in the degree of cell condensation in the construct. This aspect let the patterned HUVEC to self-assemble into a highly heterogenous capillary network, similar to what is observed *in vivo* [37]. Indeed, thicker tubular connections were established in areas of high cell condensation (ring pattern line) whereases thinner and branched tubular networks were formed in areas of low cell condensation (area outside the ring). No connections were formed by the few cells left in the central area of the ring pattern. Being limited by the predefined bioink and cell concentration, other bottom-up biofabrication processes can only offer homogeneous cell condensation within their constructs [38] or reach gradient formation by use of laborious strategies (e.g., coupling of microfluidics and direct light printing [39]).

The presence of a ring pattern in a separate layer facilitated image acquisition and analysis, providing real-time quantitative readouts of changes within the microcapillary network in response to external stimuli such as anticancer treatments. This is a major benefit compared to previous work [20,21,24,26], were mainly angiogenic sprouting was quantified. The area under the curve (AUC) and the peak's intensity, quantified from the ‘Radial profile’ signal, provided simple readouts which can measure the status of the microcapillary network. While the AUC quantification correlated with the measures of GFP-positive area, thereby providing means to trace the network growth, peak's intensity gave information about the continuity of the central microcapillary ring. Arising discontinuity of the central microcapillary ring might indicate the regression of larger established vessels in response to antiangiogenic treatment which do not only target new small angiogenic sprouting [29].

The ring microcapillary network, similar to the randomly assembled network, showed a regression of the covered area after 2 days in culture in absence of the tumor spheroid. This was expected and in accordance with previous observations reporting a similar short resistance of the microcapillary network over time in the angiogenic Matrigel assay [40]. Different to this observation, the microcapillary network growth was sustained when the STM model was assembled, suggesting that proangiogenic soluble factors supplied by the tumor spheroid were even more effective than standard pro-angiogenic culture media formulation (EGM-2). Interestingly, this effect was even more pronounced when STM models were treated with Cisplatin, the first-line drug treatment in MPM. We could measure that Cisplatin was able to reduce MPM tumor spheroids metabolic activity (Fig. S4, A), decreasing their sprouting within fibrin and increasing cell death (Fig. S4, B). Although one work reported an anti-angiogenic effect of Cisplatin, this study was performed in 2D cultures of HUVECs, grown in the presence of conditioned medium of lung cancer cells [41]. 2D systems cannot reproduce the complexity of the capillary network present in a 3D tumor that is better recapitulated by the STM reported in the present study. Indeed, in Matrigel-growing solid tumors of different origin, as well as in xenografts, Cisplatin has been reported to up-regulate VEGF and its cognate receptor VEGFR1/Flt, paradoxically favoring tumor growth and neo-angiogenesis [42]. This comparison raises warning on using traditional 2D co-cultures in studies evaluating the drug effects, because they may produce opposite results to those observed in the real tumor. The choice of an appropriate 3D model is mandatory to have a more representative picture of the tumor-vascular interactions and of the effect exerted by a specific anticancer drug on a tumor surrounded by its micro-environment.

We hypothesized that we could observe the mitigation of pro-angiogenic behavior - typical of a stressed tumor mass - by advocating the synergistic effect of anticancer-antiangiogenic drugs administrated to the model. With VEGF being the most important and best studied pro-angiogenic factor [43], we believe that its inhibition through Bevacizumab would diminish the pro-angiogenic stimuli [44]. Interestingly, our findings did not support this hypothesis when we used a Bevacizumab concentration within a commonly used range for the anticancer-antiangiogenic combination treatment. Indeed, the Bevacizumab is usually used *in vitro* at concentration from 100 ​ng⋅mL^−1^ (used to pre-treat 2D cultured HUVEC which later showed reduced tubular network formation in 3D *in vitro* angiogenesis assay [45]) to 10 ​μg⋅mL^−1^ (which was effective to induce regression of microcapillary sprouting in a 3D *in vitro* models without need to pretreat HUVEC in 2D culture [29]). Although at day 2 and 3 we observed a mild reduction of the microcapillary network after treating the STM with Bevacizumab alone at 10 ​μg⋅mL^−1^ (Fig. 5, B), this concentration did not rescue the pro-angiogenic effect induced by Cisplatin. However, additional experiments on the STM model indicated that by increasing the Bevacizumab concentration to 200 ​μg⋅mL^−1^ in the drugs combination treatment, the growth of the microcapillary network was reduced by Bevacizumab alone and that the pro-angiogenic effect of Cisplatin was abrogated (Fig. S5, A&B). Using higher Bevacizumab concentrations could result in higher *anti*-VEGF antibody levels, which could better compete with the pro-angiogenic factors being released in the model by the tumor spheroid. In support of this assumption, it has been reported that Bevacizumab administrated at a concentration of 500 ​μg⋅mL^−1^ in a perfusable model of microfluidic capillary network, stopped new capillary sprouting induced by tumor cells [24]. Additionally, the ECM-like hydrogel matrix could physically and chemically interfere with the antibody-based drugs, and only higher concentrations of Bevacizumab may reach its target when applied to STM. The same considerations may be applied to a tumor mass, when the ECM permeability may limit the delivery of Bevacizumab within the tumor bulk and the inhibition of VEGF. The anti-angiogenic effects that we obtained with high concentration of bevacizumab were not due to a reduced viability of HUVEC (Fig. S5, C). Furthermore, in line with a previous study reporting that concentrations of Bevacizumab up to 1 ​mg⋅mL^−1^ did not inhibit HUVEC viability in regular culture medium (EGM-2) [45] our transcriptome profile also showed that Bevacizumab did not modulate genes involved in HUVEC proliferation or apoptosis (Fig. S8).

In our model, Cisplatin clearly favored the HUVEC sprouting, and completely abrogated the anti-angiogenic effects of Bevacizumab. Our transcriptome profile focused on pro-angiogenic and anti-angiogenic genes, revealed that Cisplatin was an active inducer of pro-angiogenic factors (Fig. 6, A, & Fig. S8). First, it increased VEGFA, in line with a previous report [42]. Such an increase unequivocally reduces the anti-angiogenic effects of Bevacizumab that must face higher levels of its target. Second, Cisplatin induced a plethora of soluble factors known to mediate proliferation and sprouting of endothelial cells. It has already been reported that Cisplatin increases cytokines and growth factors as IL6, IL8 [46] and FGF2 [47] in solid tumors, including thoracic cancers. Besides favoring tumor spreading, these soluble factors are strong pro-angiogenic agents. Notably, some of the pro-angiogenic genes upregulated by Cisplatin synergize with VEGFA, making the anti-angiogenic efficacy of Bevacizumab harder to achieve. Other factors activate VEGFA-independent pathways: in this way, Cisplatin completely bypasses the VEGFA-inhibition elicited by Bevacizumab. We believe that the simultaneous activation of VEGFA-dependent and independent pathways elicited by Cisplatin explains the lack of any anti-angiogenic effect in STM incubated with the combination of Cisplatin and Bevacizumab.

The separate analysis of Cisplatin's effects on HUVEC/pericytes microcapillary, MPM tumor spheroid and their coculture also identified the tumor cells as the main target of Cisplatin. Indeed, MPM cells were more reactive than HUVEC cells in rewiring their transcriptome profile toward a pro-angiogenic direction (Fig. 6, Aiii). Such high pro-angiogenic *vs.* anti-angiogenic ratio may justify why Cisplatin increased endothelial cells sprouting and vessels formation in the STM (Fig. 5, A&B). When both HUVEC and tumor cells are present, as in our STM model, the balance between pro-angiogenic and anti-angiogenic factors dramatically shift toward the former upon the treatment with Cisplatin (Fig. 6, Ai&Bi). The use of STM model, including both tumor cells and capillary network, offers a reliable and precise tool to describe in depth the effects of Bevacizumab and Cisplatin in a model closer to the real tumor physiology.

To the best of our knowledge, the pro-angiogenic effect of Cisplatin in MPM has never been reported before: this is the first work describing such effect and may unveil a novel reason of the low efficacy of Cisplatin in MPM patients. The STM was also illuminating in describing the complex biological factors that may limit the efficacy of the combination therapy based on Cisplatin and Bevacizumab. Consistently, the increase in the overall survival of MPM patients treated with chemotherapy plus Bevacizumab was moderate compared with patients treated with chemotherapy alone [48]. This significant but limited benefit may be explained by the pleiotropic pro-angiogenic stimuli increased by Cisplatin that antagonize the anti-angiogenic effect of Bevacizumab.

In future prospective of this work, a system that allows for the perfusion of the microcapillary ring within the model is envisioned. Indeed, although the ring microcapillary networks showed already evidences of vessel functionality like the expression of VE-cadherin at the endothelial cell-cell junction, and presence of lumen formation (Figs. S6 and S7, Supplementary Video 1), perfusing these established networks would closely mimic function and environment of a capillary network *in vivo* [49]. Additional quantifications from the STM could be extracted as complementary readouts about tumor progression (cell migration and tumor growth) which could complete the characterization of the TME in response to treatments. Using this model, more experiments could be designed to quantify the level of proangiogenic factors released by the specific tumor spheroid and determine more effective antiangiogenic treatments. The influence of other responsible factors (e.g., Fibroblast -, Epidermal -, Insulin like -, growth factors) which compensate for the blockage of VEGF by Bevacizumab [45] could also be studied and more effective drug combinations or treatment strategies developed with the STM. Moreover, the scalability of this model could provide a platform to study not only the TME but also the crosstalk between vasculature, and a plethora of other tissues in their early stage of development and differentiation. Furthermore, this model can provide a platform for medium-to-high throughput screening of new anticancer agents or combinations.

Supplementary video related to this article can be found at https://doi.org/10.1016/j.mtbio.2022.100357

The following is the supplementary data related to this article:

Supplementary Video 1 Supplementary Video 1

## 5 Conclusions

In the reported work, we established a spatially organized multicellular 3D model which mimics the tumor microenvironment. We demonstrated that sound patterning is a simple tool to create reproducible vascular structures within a 3D *in vitro* model. We elucidated that a pre-patterned microcapillary network provides additional quantitative readouts of vascular development. Lastly, the model was validated by detecting angiogenic stimulation in presence of anticancer treatment. Altogether, this simple multicomponent model setup could be a versatile tool to investigate new promising therapeutics or patient tailored treatments, particularly in those tumors that are refractory to the current treatment and urgently need new therapeutic options.

## Author contributions

**NicolaDi Marzio**: Methodology, Investigation, Data curation, Formal analysis, Visualization, Writing – original draft, **PreetaAnanthanarayanan**: Methodology, Writing – original draft, **Anne GéraldineGuex**: Methodology, Investigation, Supervision, Writing – review & editing, **MauroAlini**: Funding acquisition, Supervision, Writing – review & editing, **ChiaraRiganti**: Conceptualization, Data curation, Funding acquisition, Supervision, Writing – review & editing, **TizianoSerra**: Conceptualization, Funding acquisition, Supervision, Writing – review & editing.

## Funding sources

The authors received funding from the European Union's 10.13039/501100007601Horizon 2020 Research and Innovation Program under grant agreement no. 860462 project PREMUROSA, the AO Research Institute Davos, the AO Development Incubator, and the AO CMF. The Department of Oncology, 10.13039/100015918University of Torino, received funding from the Italian Association of Cancer Research (10.13039/501100005010AIRC; IG21408), by 10.13039/501100009629Cassa di Risparmio di Torino Foundation (RF ​= ​2016–2443; RF2018-0568; RF ​= ​2021–0556) and by the 10.13039/100007388Compagnia di San Paolo/University of Torino Collaborative Agreement (NANOLIGHT project); ERA-Net Transcan-2-JTC 2017 (TOPMESO). This article is based upon work from 10.13039/501100000921COST Action CA17104 STRATAGEM, supported by COST (10.13039/501100000921European Cooperation in Science and Technology) (www.cost.eu).

## Declaration of competing interest

Tiziano Serra is co-founder of Mimix Biotherapeutics Ltd. (without financial support) which explores the technology in this manuscript. The company had no role in the study design, data interpretation or manuscript writing.

## References

[1] Asghar W., El Assal R., Shafiee H., Pitteri S., Paulmurugan R., Demirci U. (2015). Engineering cancer microenvironments for in vitro 3-D tumor models. Mater. Today.

[2] Rodrigues J., Heinrich M.A., Teixeira L.M., Prakash J. (2021). 3D in vitro model (R) evolution: unveiling tumor–stroma interactions. Trends in cancer.

[3] Bonelli M.A., Fumarola C., La Monica S., Alfieri R. (2017). New therapeutic strategies for malignant pleural mesothelioma. Biochem. Pharmacol..

[4] Patil N.S., Righi L., Koeppen H., Zou W., Izzo S., Grosso F., Libener R., Loiacono M., Monica V., Buttigliero C., Novello S., Hegde P.S., Papotti M., Kowanetz M., Scagliotti G.V. (2018). Molecular and histopathological characterization of the tumor immune microenvironment in advanced stage of malignant pleural mesothelioma. J. Thorac. Oncol..

[5] S. Thellung, R. Würth, R.E. Favoni, M. Nizzari, A. Pattarozzi, A. Daga, T. Florio, F. Barbieri, S. Farmacologia, M. Interna, I. Aou, S. Martino, V.B. Xv, Molecular Pharmacology of Malignant Pleural Mesothelioma : Challenges and Perspectives from Preclinical and Clinical Studies, 10.2174/1389450116666150804110714. 26240051

[6] Tsao A.S., Wistuba I., Roth J.A., Kindler H.L. (2009). Malignant pleural mesothelioma. J. Clin. Oncol..

[7] Munn L.L. (2003). Aberrant vascular architecture in tumors and its importance in drug-based therapies. Drug Discov. Today.

[8] Chu G.J., Zandwijk N.V., Rasko J.E.J. (2019). The immune microenvironment in mesothelioma : mechanisms of resistance to immunotherapy. Front. Oncol..

[9] Nabavi N., Bennewith K.L., Churg A., Wang Y., Collins C.C., Mutti L. (2016). Switching off malignant mesothelioma: exploiting the hypoxic microenvironment. Genes and Cancer.

[10] Riganti C., Doublier S., Aldieri E., Orecchia S., Betta P.G., Gazzano E., Ghigo D., Bosia A. (2008). Asbestos induces doxorubicin resistance in MM98 mesothelioma cells <em>via</em> HIF-1α. Eur. Respir. J..

[11] Carmeliet P., Jain R.K. (2000). Angiogenesis in cancer and other diseases. Nature.

[12] Dong H., Guo H., Xie L., Wang G., Zhong X., Khoury T., Tan D., Zhang H. (2013). The metastasis-associated gene MTA3, a component of the mi-2/NuRD transcriptional repression complex, predicts prognosis of gastroesophageal junction adenocarcinoma. PLoS One.

[13] Morishita Y., Ookawara S., Hirahara I., Muto S., Nagata D. (2016). HIF-1alpha mediates Hypoxia-induced epithelial-mesenchymal transition in peritoneal mesothelial cells. Ren. Fail..

[14] Bhujwalla Z.M., Artemov D., Aboagye E., Ackerstaff E., Gillies R.J., Natarajan K., Solaiyappan M. (2001).

[15] Ingthorsson S., Sigurdsson V., Fridriksdottir A.J., Jonasson J.G., Kjartansson J., Magnusson M.K., Gudjonsson T. (2010). Endothelial cells stimulate growth of normal and cancerous breast epithelial cells in 3D culture. BMC Res. Notes.

[16] Chiew G.G.Y., Fu A., Perng Low K., Qian Luo K. (2015). Physical supports from liver cancer cells are essential for differentiation and remodeling of endothelial cells in a HepG2-HUVEC co-culture model. Sci. Rep..

[17] Ou J., Guan D., Yang Y. (2019). Non-contact co-culture with human vascular endothelial cells promotes epithelial-to-mesenchymal transition of cervical cancer SiHa cells by activating the NOTCH1/LOX/SNAIL pathway. Cell. Mol. Biol. Lett..

[18] Wang X., Sun Q., Pei J. (2018). Microfluidic-based 3D engineered microvascular networks and their applications in vascularized microtumor models. Micromachines.

[19] Zervantonakis I.K., Hughes-Alford S.K., Charest J.L., Condeelis J.S., Gertler F.B., Kamm R.D. (2012). Three-dimensional microfluidic model for tumor cell intravasation and endothelial barrier function. Proc. Natl. Acad. Sci. USA.

[20] Lee S., Kim S., Koo D.-J., Yu J., Cho H., Lee H., Song J.M., Kim S.-Y., Min D.-H., Jeon N.L. (2021). 3D microfluidic platform and tumor vascular mapping for evaluating anti-angiogenic RNAi-based nanomedicine. ACS Nano.

[21] Nashimoto Y., Okada R., Hanada S., Arima Y., Nishiyama K., Miura T., Yokokawa R. (2020). Vascularized cancer on a chip: the effect of perfusion on growth and drug delivery of tumor spheroid. Biomaterials.

[22] Kim C., Kasuya J., Jeon J., Chung S., Kamm R.D. (2015). A quantitative microfluidic angiogenesis screen for studying anti-angiogenic therapeutic drugs. Lab Chip.

[23] Jeon J.S., Bersini S., Gilardi M., Dubini G., Charest J.L., Moretti M., Kamm R.D. (2015). Human 3D vascularized organotypic microfluidic assays to study breast cancer cell extravasation. Proc. Natl. Acad. Sci. USA.

[24] Lee H., Park W., Ryu H., Jeon N.L. (2014). A microfluidic platform for quantitative analysis of cancer angiogenesis and intravasation. Biomicrofluidics.

[25] Rajasekar S., Lin D.S.Y., Abdul L., Liu A., Sotra A., Zhang F., Zhang B. (2020). IFlowPlate—a customized 384-well plate for the culture of perfusable vascularized colon organoids. Adv. Mater..

[26] Li Q., Niu K., Wang D., Xuan L., Wang X. (2022). Low-cost rapid prototyping and assembly of an open microfluidic device for a 3D vascularized organ-on-a-chip. Lab Chip.

[27] Guex A.G., Di Marzio N., Eglin D., Alini M., Serra T. (2021). The waves that make the pattern: a review on acoustic manipulation in biomedical research. Mater Today Bio.

[28] Petta D., Basoli V., Pellicciotta D., Tognato R., Barcik J., Arrigoni C., Della Bella E., Armiento A.R., Candrian C., Richards R.G. (2020). Sound-induced morphogenesis of multicellular systems for rapid orchestration of vascular networks. Biofabrication.

[29] Brennen W.N., Nguyen H., Dalrymple S.L., Reppert-Gerber S., Kim J., Isaacs J.T., Hammers H. (2016). Assessing angiogenic responses induced by primary human prostate stromal cells in a three-dimensional fibrin matrix assay. Oncotarget.

[30] Armstrong J.P., Puetzer J.L., Serio A., Guex A.G., Kapnisi M., Breant A., Zong Y., Assal V., Skaalure S.C., King O. (2018). Engineering anisotropic muscle tissue using acoustic cell patterning. Adv. Mater..

[31] Kang B., Shin J., Park H.-J., Rhyou C., Kang D., Lee S.-J., Yoon Y.-s., Cho S.-W., Lee H. (2018). High-resolution acoustophoretic 3D cell patterning to construct functional collateral cylindroids for ischemia therapy. Nat. Commun..

[32] Bouyer C., Chen P., Güven S., Demirtaş T.T., Nieland T.J., Padilla F., Demirci U. (2016). A bio-acoustic levitational (BAL) assembly method for engineering of multilayered, 3D brain-like constructs, using human embryonic stem cell derived neuro-progenitors. Adv. Mater..

[33] Naseer S.M., Manbachi A., Samandari M., Walch P., Gao Y., Zhang Y.S., Davoudi F., Wang W., Abrinia K., Cooper J.M. (2017). Surface acoustic waves induced micropatterning of cells in gelatin methacryloyl (GelMA) hydrogels. Biofabrication.

[34] Kopecka J., Salaroglio I.C., Righi L., Libener R., Orecchia S., Grosso F., Milosevic V., Ananthanarayanan P., Ricci L., Capelletto E., Pradotto M., Napoli F., Di Maio M., Novello S., Rubinstein M., Scagliotti G.V., Riganti C. (2018). Loss of C/EBP-β LIP drives cisplatin resistance in malignant pleural mesothelioma. Lung Cancer.

[35] Song H.-H.G., Park K.M., Gerecht S. (2014). Hydrogels to model 3D in vitro microenvironment of tumor vascularization. Adv. Drug Deliv. Rev..

[36] Ren T., Chen P., Gu L., Ogut M.G., Demirci U. (2020). Soft ring-shaped cellu-robots with simultaneous locomotion in batches. Adv. Mater..

[37] Nebuloni L., Kuhn G.A., Vogel J., Müller R. (2014). A novel in vivo vascular imaging approach for hierarchical quantification of vasculature using contrast enhanced micro-computed tomography. PLoS One.

[38] Heinrich M.A., Liu W., Jimenez A., Yang J., Akpek A., Liu X., Pi Q., Mu X., Hu N., Schiffelers R.M., Prakash J., Xie J., Zhang Y.S. (2019). 3D bioprinting: from benches to translational applications. Small.

[39] Wang M., Li W., Mille L.S., Ching T., Luo Z., Tang G., Garciamendez C.E., Lesha A., Hashimoto M., Zhang Y.S. (2022). Digital light processing based bioprinting with composable gradients. Adv. Mater..

[40] Khoo C.P., Micklem K., Watt S.M. (2011). A comparison of methods for quantifying angiogenesis in the Matrigel assay in vitro. Tissue Eng. C Methods.

[41] Ramer R., Schmied T., Wagner C., Haustein M., Hinz B. (2018). The antiangiogenic action of cisplatin on endothelial cells is mediated through the release of tissue inhibitor of matrix metalloproteinases-1 from lung cancer cells. Oncotarget.

[42] Tsuchida R., Das B., Yeger H., Koren G., Shibuya M., Thorner P., Baruchel S., Malkin D. (2008). Cisplatin treatment increases survival and expansion of a highly tumorigenic side-population fraction by upregulating VEGF/Flt1 autocrine signaling. Oncogene.

[43] Shibuya M. (2008). Vascular endothelial growth factor-dependent and-independent regulation of angiogenesis. BMB reports.

[44] Park S.A., Jeong M.S., Ha K.-T., Jang S.B. (2018). Structure and function of vascular endothelial growth factor and its receptor system. BMB reports.

[45] Liu Y., Tian H., Blobe G.C., Theuer C.P., Hurwitz H.I., Nixon A.B. (2014). Effects of the combination of TRC105 and bevacizumab on endothelial cell biology. Invest. N. Drugs.

[46] Kiss E., Abdelwahab E.H.M.M., Steib A., Papp E., Torok Z., Jakab L., Smuk G., Sarosi V., Pongracz J.E. (2020). Cisplatin treatment induced interleukin 6 and 8 production alters lung adenocarcinoma cell migration in an oncogenic mutation dependent manner. Respir. Res..

[47] Nakawatari M., Iwakawa M., Ohno T., Katoh S., Tamaki T., Imadome K., Sakai M., Tsujii H., Imai T. (2007). Chemoradiation-induced expression of fibroblast growth factor-2 and laminin in patients with cervical cancer. Cancer Biol. Ther..

[48] Zalcman G., Mazieres J., Margery J., Greillier L., Audigier-Valette C., Moro-Sibilot D., Molinier O., Corre R., Monnet I., Gounant V. (2016). Bevacizumab for newly diagnosed pleural mesothelioma in the Mesothelioma Avastin Cisplatin Pemetrexed Study (MAPS): a randomised, controlled, open-label, phase 3 trial. Lancet.

[49] Van Duinen V., Zhu D., Ramakers C., Van Zonneveld A., Vulto P., Hankemeier T. (2019). Perfused 3D angiogenic sprouting in a high-throughput in vitro platform. Angiogenesis.

