# Supplementary material for: Sound-based assembly of a microcapillary network in a saturn-like tumor model for drug testing
Source: Mater Today Bio. 2022 Jul 12;16:100357. doi: 10.1016/j.mtbio.2022.100357 (PMC9307464; doi:10.1016/j.mtbio.2022.100357)
Supplement: Multimedia component 3 [file mmc3.docx]

**Supplementary Material**

**Sound based assembly of a microcapillary network in a Saturn-like tumor model for drug testing**

N. Di Marzio^1,2^, P. Ananthanarayanan^3^, A.G. Guex^1^, M. Alini^1^, C. Riganti^3,4^, T. Serra^1^

^1^ AO Research Institute Davos, 7270 Davos, Switzerland

^2^ Department of Health Sciences, Università del Piemonte Orientale (UPO), Novara, Italy

^3^ Department of Oncology, University of Torino, 10126, Torino, Italy

^4^ Inter-departmental Centre “G. Scansetti” for Studies on Asbestos and other Toxic Particulates, University of

Torino, 10126, Torino, Italy


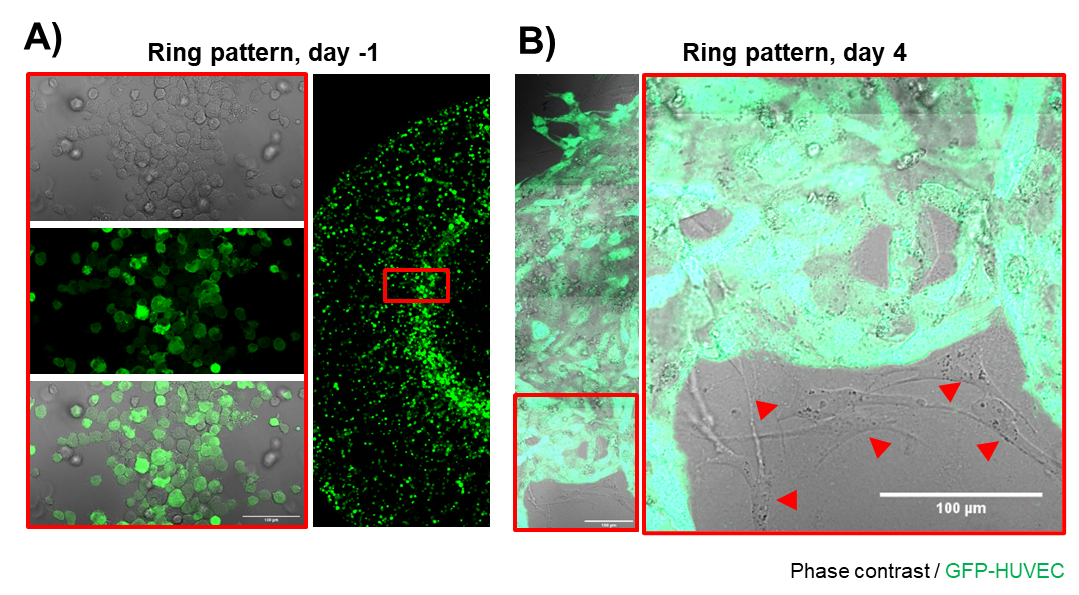


**Figure S1:** **Sound patterned cell condensation and alignment of pericytes along the contour of the GFP-HUVEC capillaries.** A) The sound patterned cells had directed contact after the patterning procedure, HUVEC and pericytes at 20:1 cell/cell ration were randomly positioned within the pattern line. B) At day 4 the pericytes (red arrows) were observed aligned along the contour of the GFP-HUVEC capillaries


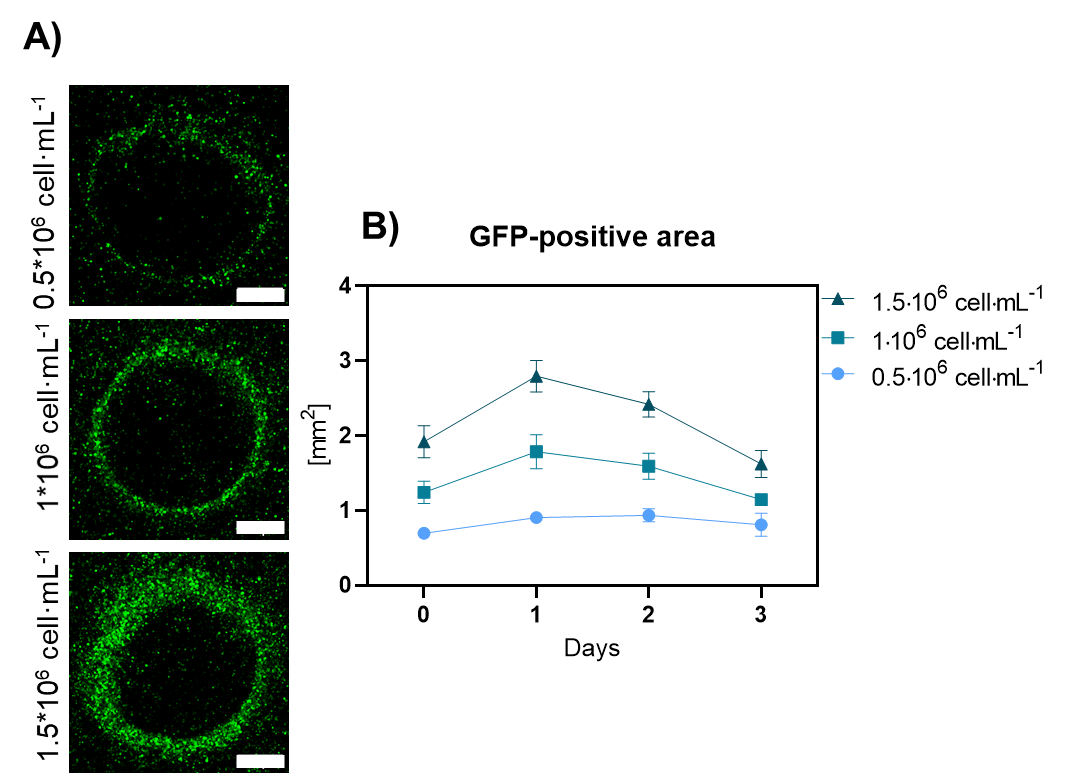


**Figure S2:** **Cell concentration optimization for sound patterning.** A) Fibrin gels with a cell concentration of 0.5, 1 or 1.5·10^6^ cell·mL^-1^ were tested and the sound patterning generated rings with different thickness but same diameter. B) During the culture time, the GFP-positive area was proportional to the cell concentration used for sound patterning.


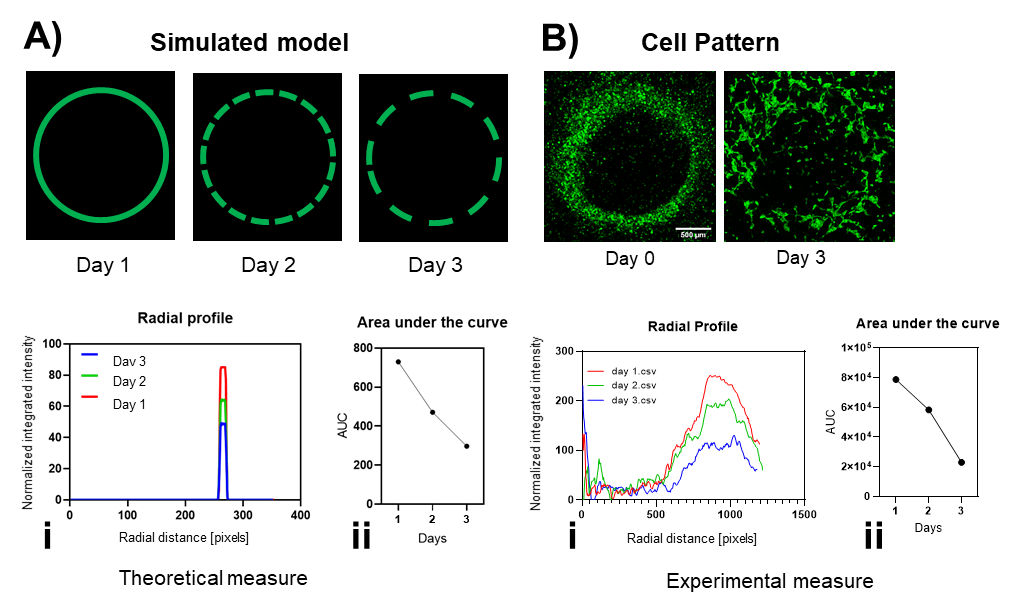


**Figure S3: 'Radial profile' peak maximum intensity is proportional to the continuity of the ring pattern.** A) Theoretical measure of a simulated model image was used to test the correlation between intensity value of the peak maximum and the continuity of the ring shape. B) An experimental measure on cell pattern images was used to validate their correlation.


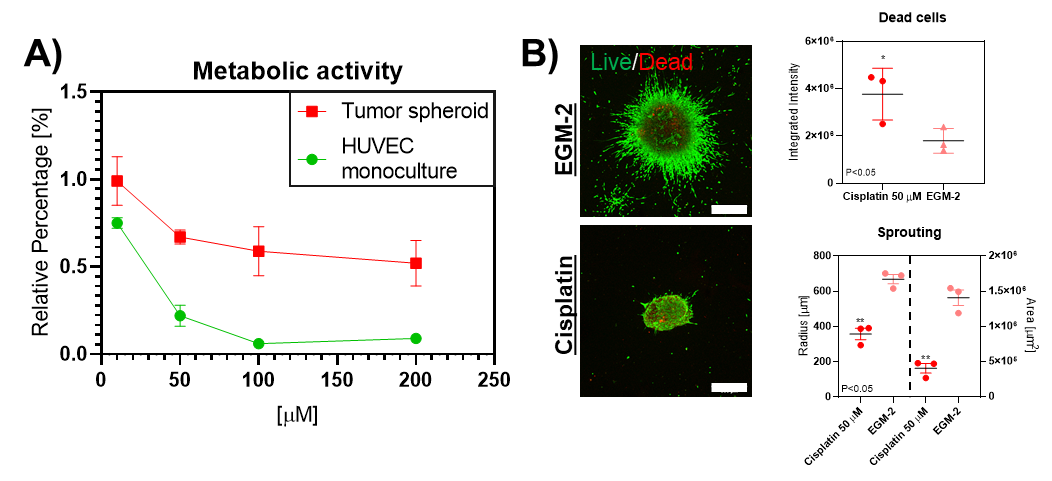


**Figure S4:** **Cisplatin treatment effect on tumor spheroid and HUVEC.** A) Metabolic activity of heterotypic tumor spheroids (MPM:MRC-5 in a 10:1 cell ratio) and HUVEC monoculture (5000 cells/well) after 48 hours of incubation with multiple Cisplatin concentrations (0-200 µM), Cisplatin 50 µM was extracted as treatment concentration for the STM drug tests. B) Live/Dead assay (Calcein AM / Ethidium homodimer) was performed on the tumor spheroid in fibrin, after 4 days of treatment with Cisplatin 50 µM. 2-fold increase level of dead cells and limited sprouting were registered after cisplatin administration. n=3. Scale bars 500 µm.


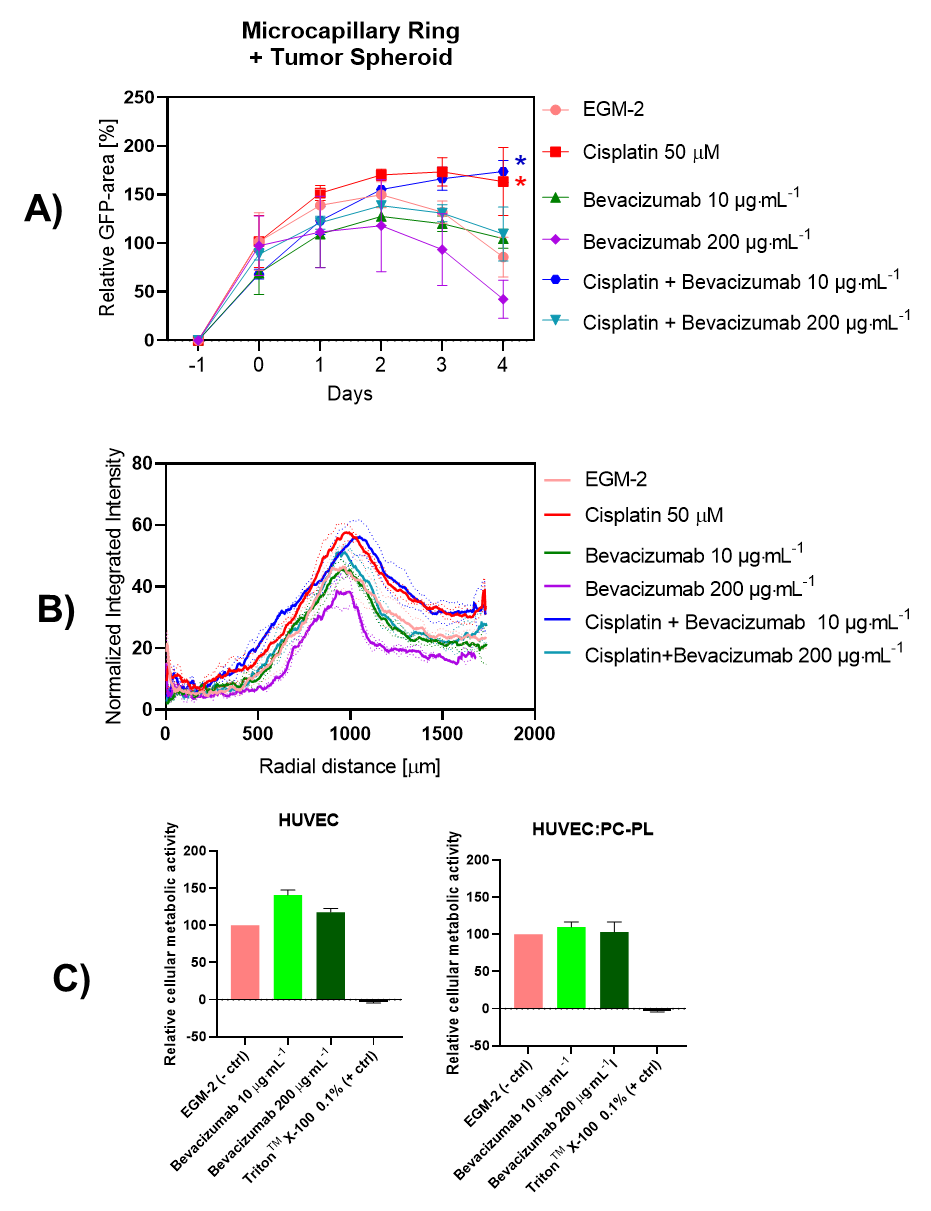


**Figure S5: Growth of the ring microcapillary network is reduced with higher Bevacizumab concentration.** A) The growth trend of GFP-HUVEC network’s area over time indicates that the presence of the tumor spheroid is able to stimulate the microcapillary network growth. When the STM is treated with the anticancer drug (Cisplatin 50 µM), Bevacizumab (10 or 200 µg·mL^-1^), or their combination (Cisplatin 50 µM- Bevacizumab 10 µg·mL^-1^), it induces the ring microcapillary network to cover 50% more area compared to the EGM-2 control condition. When it is treated with Cisplatin 50 µM- Bevacizumab 200 µg·mL^-1^, the ring microcapillary network area is comparable to the EGM-2 treated condition. N=2 and n=3. B) Similar response to treatments emerged from the radial profile analysis. Continuous lines indicate mean value, dotted lines indicate SD. C) Cell metabolic activity of HUVEC or HUVEC:PC 20:1 on Tissue Culture Treated Polystyrene (TCPS, 2D) was not affected by either the 10 or the 200 µg·mL^-1^ Bevacizumab treatments concentration. n=3.


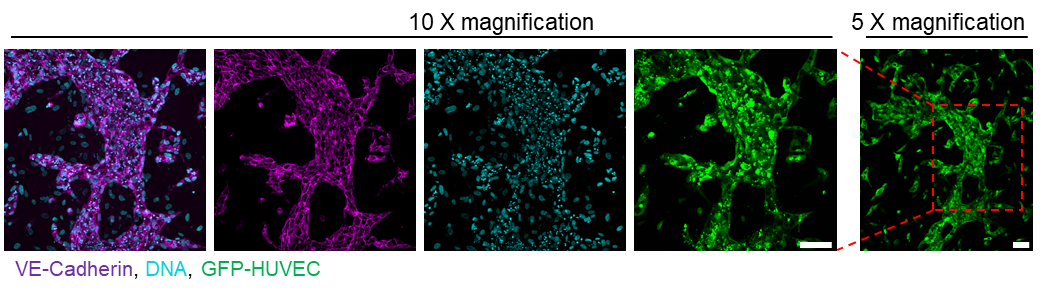


**Figure S6: VE-cadherin expression in microcapillary network patterns.** Immunofluorescence staining HUVEC:Perycites of ring microcapillary networks demonstrates VE-cadherin expression at the endothelial cell-cell junction. Representative images of 3 separate samples. Scale bars 100 µm.


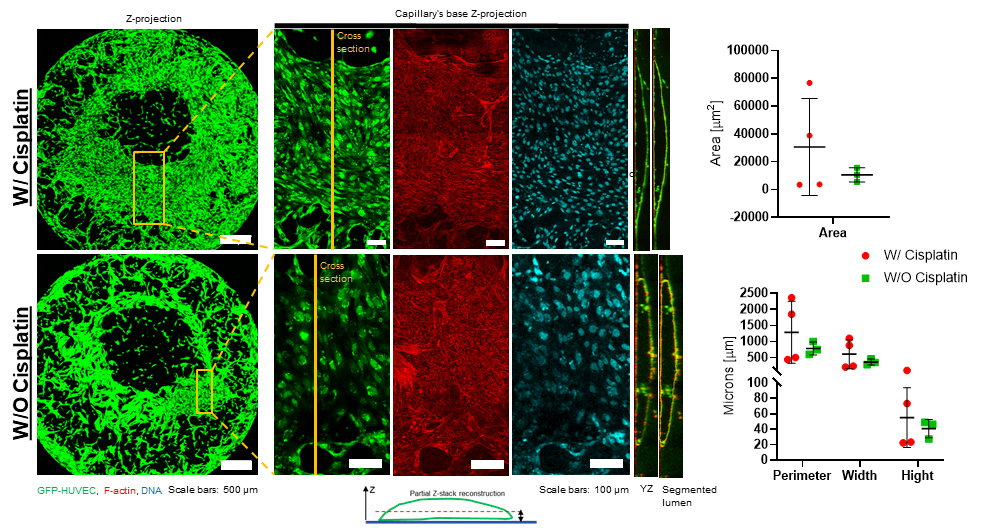


**Figure S7: Lumen measure in the microcapillary of the STM model w/ or w/o Cisplatin treatment.** Based on ImageJ analysis the lumen dimensions were measured. In presence of Cisplatin treatment, the microcapillary rings contained bigger and more continuous lumen. The endothelial cells lining in the capillary walls were found to be aligned into preferential directions and have elongated morphology under Cisplatin treatment in comparison with their usual polygonal morphology, known indicator of increased levels of VEGF.


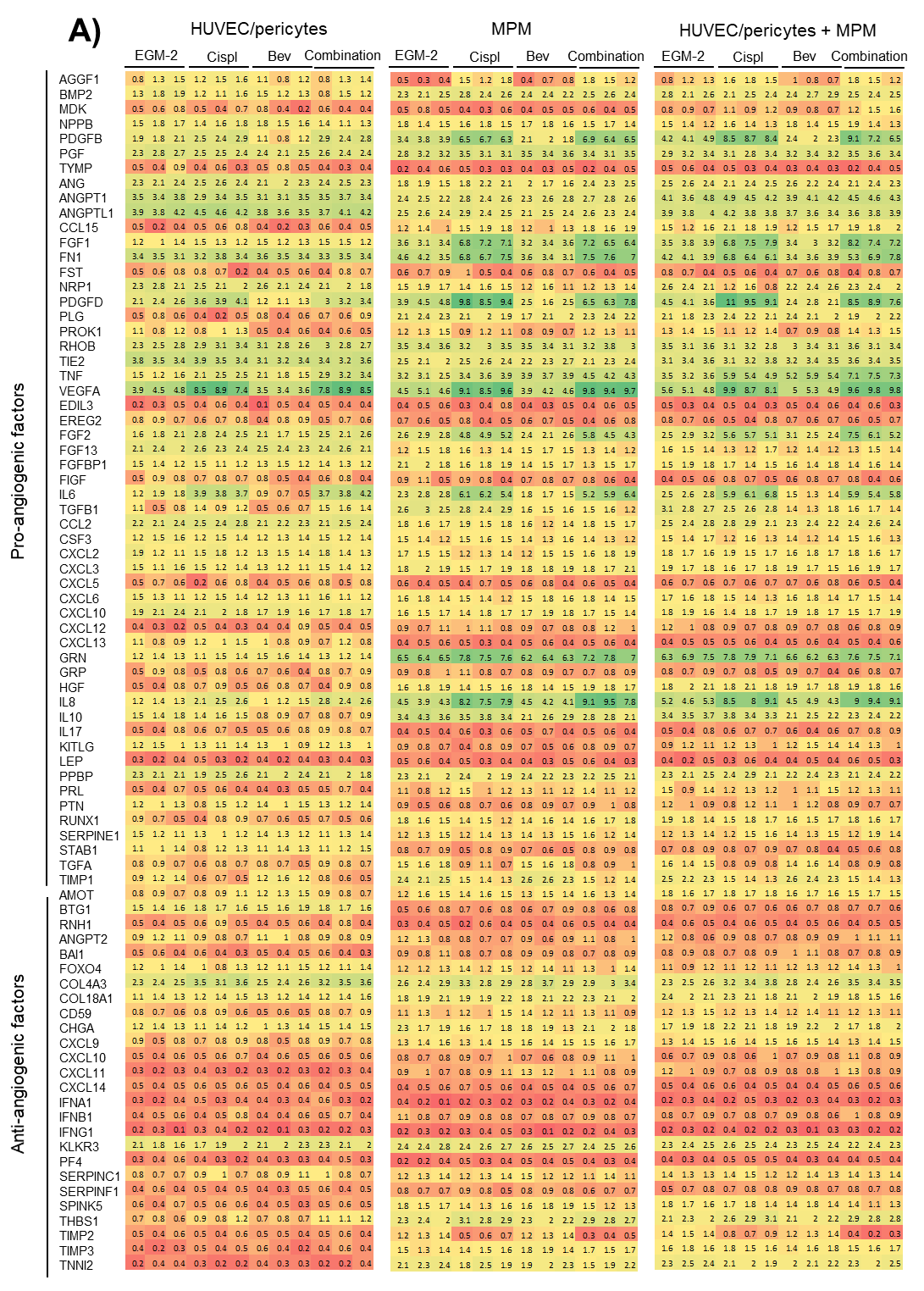


**Figure S8: Profile of angiogenesis-related genes expression in vascular bed, malignant pleural mesothelioma spheroid, and their coculture upon treatments.** (A) Heatmap of pro-angiogenic and anti-angiogenic genes in HUVEC-pericytes, (B) malignant pleural mesothelioma spheroids and (C) their coculture grown for 4 days in fresh medium (EGM-2) or treated with cisplatin (Cispl, 50 µM), Bevacizumab (Bev, 10 µg·mL^-1^) or their combination (Combination), evaluated by a PCR-array. The numbers in each cell indicate the relative expression of each gene, normalized on the mean expression of the housekeeping genes B2M, GAPDH, HPRT1, TBP, RLP0, GUSB (Gene Expression Quantitation software, Bio-Rad Laboratories). N=3, n=2. The whole statistical analysis is reported in the Supplementary File 1.
